# The magnitude of LFA-1/ICAM-1 forces fine-tune TCR-triggered T cell activation

**DOI:** 10.1126/sciadv.abg4485

**Published:** 2022-02-25

**Authors:** Victor Pui-Yan Ma, Yuesong Hu, Anna V. Kellner, Joshua M. Brockman, Arventh Velusamy, Aaron T. Blanchard, Brian D. Evavold, Ronen Alon, Khalid Salaita

**Affiliations:** 1 Department of Chemistry, Emory University, Atlanta, GA 30322, USA.; 2 Wallace H. Coulter Department of Biomedical Engineering, Georgia Institute of Technology, Emory University, Atlanta, GA 30332, USA.; 3 Division of Microbiology and Immunology, Department of Pathology, University of Utah, Salt Lake City, UT 84112, USA.; 4 Department of Immunology, The Weizmann Institute of Science, Rehovot 7610001, Israel.; 5 Winship Cancer Institute, Emory University, Atlanta, GA 30322, USA.

## Abstract

T cells defend against cancer and viral infections by rapidly scanning the surface of target cells seeking specific peptide antigens. This key process in adaptive immunity is sparked upon T cell receptor (TCR) binding of antigens within cell-cell junctions stabilized by integrin (LFA-1)/intercellular adhesion molecule–1 (ICAM-1) complexes. A long-standing question in this area is whether the forces transmitted through the LFA-1/ICAM-1 complex tune T cell signaling. Here, we use spectrally encoded DNA tension probes to reveal the first maps of LFA-1 and TCR forces generated by the T cell cytoskeleton upon antigen recognition. DNA probes that control the magnitude of LFA-1 force show that *F*>12 pN potentiates antigen-dependent T cell activation by enhancing T cell–substrate engagement. LFA-1/ICAM-1 mechanical events with *F*>12 pN also enhance the discriminatory power of the TCR when presented with near cognate antigens. Overall, our results show that T cells integrate multiple channels of mechanical information through different ligand-receptor pairs to tune function.

## Teaser

DNA-based tension probes reveal that the magnitude of LFA-1 forces tune T cell activation.

## INTRODUCTION

The specific recognition of the peptide-loaded major histocompatibility complex (pMHC) by the T cell receptor (TCR) triggers initial T cell activation, yet the intensity of activation, and subsequent T cell function and developmental fate, is fine-tuned by additional ligations of multiple co-receptors, such as lymphocyte function-associated antigen–1 (LFA-1), CD28, cytotoxic T-lymphocyte associated protein 4, and programmed cell death protein 1 (*1*). Among these different co-receptors, the T cell integrin LFA-1 (α_L_β_2_) plays a critical role in physically stabilizing the junction between T cells and their target antigen-presenting cells (APCs) (*2*–*4*). In response to antigen stimulation of the TCR, the LFA-1 heterodimer adopts a high-affinity state (*5*) that recognizes the broadly expressed cell adhesion molecule intercellular adhesion molecule–1 (ICAM-1) to form the LFA-1/ICAM-1 complex at the cell-cell junction (*6*). This complex stabilizes transient T cell–APC interactions and hence sustains T cell activation (*7*). Further underscoring this point, T cells that cannot form LFA-1/ICAM-1 interactions have defective signaling capability and hampered function (*8*–*10*). Accordingly, understanding the molecular inputs detected by LFA-1 is fundamental to improving our understanding of the adaptive immune system.

Given the role of LFA-1/ICAM-1 in mediating T cell adhesion, it has long been suspected that this ligand-receptor complex transmits and experiences forces as a part of normal T cell function (*11*). In support of this view, constraining ICAM-1 mobility contributes to enhancement of T cell and natural killer (NK) cell function (*12*, *13*). Furthermore, LFA-1 binding to ICAM-1 is diminished if ICAM-1 is soluble, which is in contrast to immobile ICAM-1 that induces ligand-receptor binding and strong cell adhesion (*14*). Most recently, Springer and others (*15*) created a genetically encoded tension sensor (GETS) and showed that the intracellular LFA-1 β_2_ subunit experiences ~2 pN of force at the leading edge of migrating Jurkat lymphoblast. Together, prior work strongly suggests that ICAM-1 experiences piconewton forces transmitted through LFA-1. That said, the specific magnitude, timing, and location of LFA-1/ICAM-1 forces and the functional roles of these forces in tuning T cell activation and function remain unknown. Are the ~2-pN forces at the β_2_ intracellular domain transmitted across the plasma membrane and delivered to ICAM-1? More broadly, given that the TCR and LFA-1 receptors differentially engage cytoskeletal elements in the same cell, how are TCR and LFA-1 forces coordinated, and do these forces cooperate to mediate T cell activation and function?

Here, we use molecular tension probes to control and measure LFA-1 forces and reveal that the precise piconewton magnitude of tension fine-tunes TCR signaling and T cell function. We achieve our work in the ovalbumin (OVA) primary T cell model (OT-1) rather than using immortalized cell lines that display aberrant signaling and mechanics (*16*–*19*). We find that LFA-1–mediated T cell contact, adhesion, and spreading are controlled by the lateral mobility of ICAM-1, thus demonstrating that LFA-1/ICAM-1 forces enhance T cell spreading. Using high-resolution DNA-based tension probes, we show that the LFA-1/ICAM-1 complex experiences forces equal to or greater than 5 pN, with a small subset of LFA-1 transmitting forces that exceed 19 pN at the periphery of actively spreading T cells. Unexpectedly, these values are one order of magnitude greater than estimates obtained using GETS probes. Furthermore, multiplexed spectrally encoded tension probes reveal that TCR and LFA-1 forces are spatially distinct likely because of different mechanisms of engagement to the cytoskeleton. DNA tension gauge tethers (TGTs) (*20*), which manipulate LFA-1 forces, demonstrate that LFA-1 forces potentiate TCR activation and enhance the sensitivity of the T cell response to antigen. TCR and LFA-1 mechanics are coupled, as low pMHC antigen density was found to enhance the rate of mechanical sampling of LFA-1 compared to high-density antigen stimulation. Our results therefore suggest that LFA-1 and the TCR each decode mechanical information, which is integrated and transduced by the cell.

## RESULTS

### ICAM-1 mobility tunes antigenic T cell signaling and spreading

A well-established experimental system to study T cell response to antigen is the supported lipid bilayer (SLB), which is a two-dimensional assembly of lipids that forms on a glass slide. The SLB is useful because it is composed of phospholipids and proteins and displays lateral mobility that captures some of the physical and chemical properties of the cell membrane (*21*). The lateral mobility of ligands within the plasma membrane is important as the mobilities of both pMHC and other auxiliary ligands such as ICAM-1 have been shown to influence the strength of T cell activation. For example, seminal work by Groves, Dustin and others (*22*) demonstrated that SLBs physically impeded by nanopatterned grids enhanced T cell activation by trapping the signaling microclusters at the periphery of the T cell surface junction. Burkhardt and others (*13*) showed that dendritic cell maturation leads to slowed lateral diffusion of ICAM-1 (*D* = ~0.025 to 0.04 μm^2^/s) and this reduced mobility enhanced T cell adhesion and activation. Sensitivity to ICAM-1 was also shown in NK cells as demonstrated by Long and others (*12*). Given prior work showing that the lateral mobility of ligands is an important biophysical parameter that controls T cell activation, we first aimed to use the SLB platform to tune ligand mobility in a controlled manner and test how the mobilities of pMHC and ICAM-1 regulate early T cell signaling and adhesion.

We used two types of phospholipids to generate SLBs with differential membrane mobility: 1,2-dioleoyl-*sn*-glycero-3-phosphocholine (DOPC) lipids with two degrees of unsaturation and hence highly mobile at room temperature (RT), and 1,2-dipalmitoyl-*sn*-glycero-3-phosphocholine (DPPC), a fully saturated phospholipid that is in the gel phase at RT. The cognate pMHC antigen (SIINFEKL peptide derived from OVA, OVA-N4) along with an engineered ICAM-1 (truncated, C-terminal biotinylated and green fluorescent protein (GFP)–modified, Fc–sfGFP–ICAM-1; fig. S1 and table S1) were tethered to the SLB via biotin-streptavidin interaction. The density of ICAM-1 was ~800 molecules/μm^2^ (fig. S2 and table S2) on these SLBs, which is within the physiological range found on APCs and activated endothelial cells (200 to 1600 molecules/μm^2^) (*23*–*25*). Ligands exhibited apparent diffusion coefficients of ~1 μm^2^/s on DOPC bilayers and were negligible on DPPC bilayers (fig. S3). Thus, DOPC and DPPC SLBs represent chemically similar surfaces that drastically differ in their lateral mechanical properties, reflecting two extremes in ligand mobility. In the same experiment, antigen densities were varied by ~100-fold (by titration). T cell responses were determined at 30 min after surface engagement by spreading and immunostaining of phosphorylated zeta chain of T Cell receptor associated protein kinase 70 (pY-ZAP70), a canonical marker of proximal TCR signaling. At low antigen density (~0.8 molecules/μm^2^), ligands tethered to DOPC bilayers enabled efficient T cell spreading and high pY-ZAP70 levels, while cell interaction with the corresponding DPPC bilayers were limited (fig. S4). At higher pMHC density (~80 molecules/μm^2^), we observed comparable spreading and pY-ZAP70 responses regardless of the ligand mobilities (fig. S4). On fluid DOPC membranes, LFA-1/ICAM-1 interaction was critical, as cells showed poor spreading and activation in response to pMHC without surface-tethered ICAM-1 (fig. S4). On DPPC membranes, LFA-1/ICAM-1 seemed to be less critical, as cells showed spreading and activation on pMHC surface lacking tethered ICAM-1. Overall, this observation is in line with literature precedent showing that laterally restrained ligands favor T cell signaling.

**Fig. 1.**
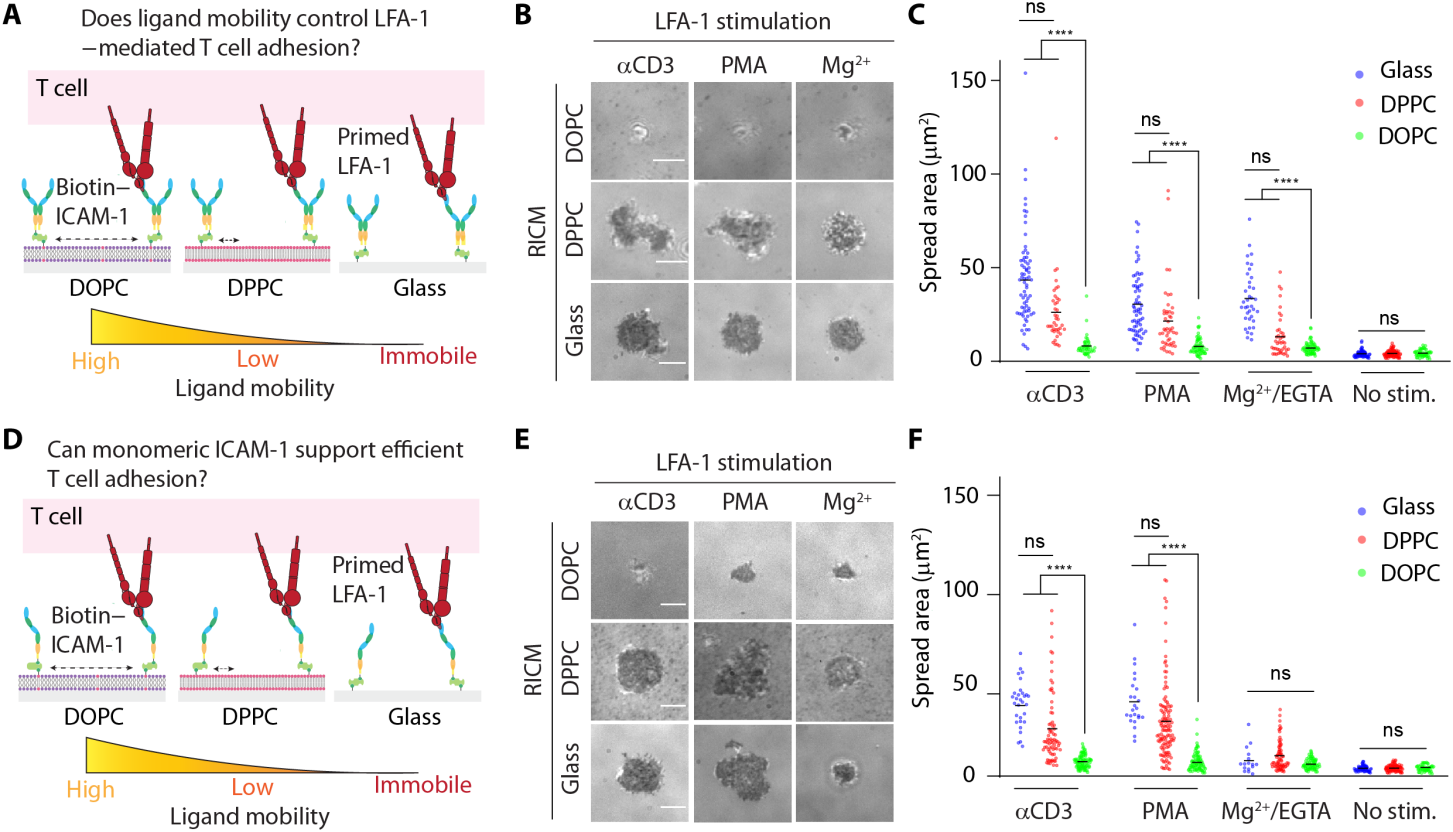
LFA-1–dependent T cell spreading favors low-mobility ICAM-1. (**A**) In vitro reconstitution of LFA-1/ICAM-1 interaction in lipid bilayers with either low (DPPC) or high (DOPC) lateral mobility using dimeric ICAM-1. (**B**) RICM images showing LFA-1–primed naïve OT-1 cells spreading on substrate after ~30 min of seeding. (**C**) Plot quantifying the spread area of cells on these substrates after stimulation with different agents. (**D**) In vitro reconstitution of LFA-1/ICAM-1 interaction in lipid bilayers with either low (DPPC) or high (DOPC) lateral mobility using monomeric ICAM-1. (**E**) RICM images showing LFA-1–primed naïve OT-1 cells spreading on substrate after ~30 min of seeding. (**F**) Plot quantifying the spread area of cells on these substrates after stimulation with different agents. Density of ICAM-1 is ~800 molecules/μm^2^ on both surfaces. *n* > 40 cells from three different experiments. Line represents mean. ****P < 0.0001. Scale bars, 5 μm. ns, not significant.

We next asked if the enhanced T cell spreading and activation is a result of LFA-1/ICAM-1 binding and whether this interaction alone is affected by ICAM-1 mobility. To this end, we prepared SLBs presenting only the dimeric ICAM-1 to decouple contributions from tethered TCR/pMHC interactions that could function to enhance cell adhesion. Because naïve cells do not respond to ICAM-1 without LFA-1 priming, we plated the cells on ICAM-1–coated surfaces and simultaneously activated cells with αCD3ε monoclonal antibody or phorbol 12-myristate 13-acetate (PMA). These two reagents are known to promote inside-out activation of LFA-1, allowing the receptor to bind ICAM-1 following the onset of intracellular signaling. We also used Mg^2+^/EGTA to conformationally change LFA-1 into a high-affinity state, bypassing the inside-out signaling. Upon incubating naïve T cells with these ICAM-1–presenting surfaces in the presence of the stimulus for 30 min, we analyzed T cell adhesion using reflection interference contrast microscopy (RICM) (Fig. 1). All stimuli failed to initiate LFA-1–mediated spreading on the fluid DOPC bilayer. In contrast, ICAM-1 tethered to the DPPC bilayer supported strong T cell spreading. Further validating this result, ICAM-1 immobilized on glass coverslips initiated equally efficient T cell adhesion (Fig. 1, A to C). Because ICAM-1 has been reported to exist in different oligomerization states including as a dimer and monomer in the cell membrane (*26*–*28*), we also engineered and tested a monomeric ICAM-1 (fig. S1 and table S1). This ligand generated nearly identical results as that of the dimeric ICAM-1, with the exception that Mg^2+^/EGTA simulation failed to trigger T cell spreading on glass (Fig. 1, D to F). Overall, these results indicate that laterally mobile ICAM-1 fails to promote T cell adhesion in the absence of membrane-bound antigen. Thus, these initial experiments show an important role for LFA-1/ICAM-1 mechanical resistance in mediating cell spreading. This led us to next investigate the magnitude, timing, and spatial distribution of LFA-1 forces transmitted to ICAM-1.

### DNA tension sensors reveal that LFA-1/ICAM-1 bonds experience 4.7- to 19-pN forces following T cell stimulation

**Fig. 2.**
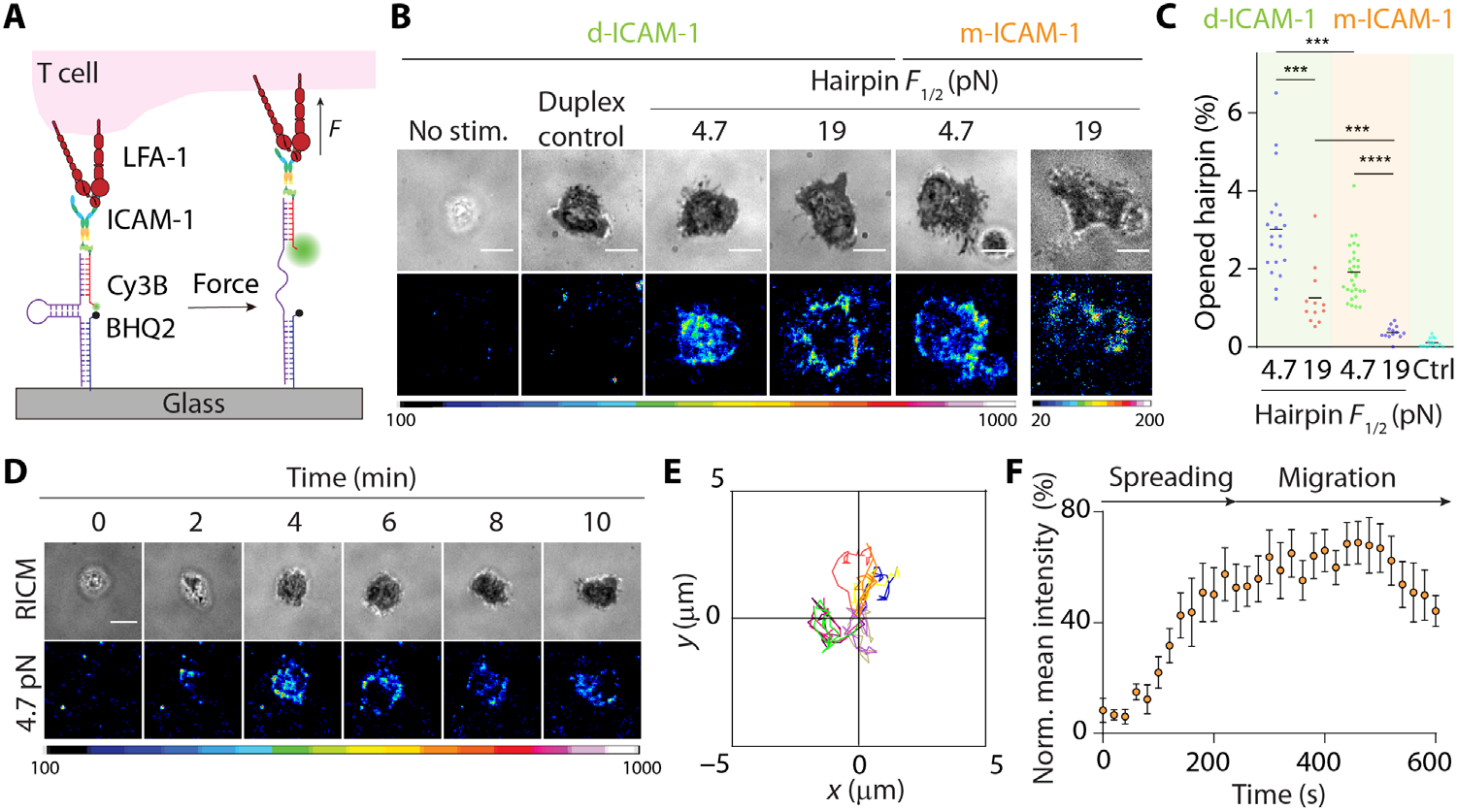
Extracellular DNA-based LFA-1 tension probes. (**A**) Design of covalently grafted ICAM-1 DNA-based tension probes. (**B**) RICM and tension images show that αCD3ε-treated naïve OT-1 cells spread and generated tension on probes with *F*_1/2_ of 4.7 or 19 pN after ~15 to 20 min of plating. Controls include nonprimed naïve OT-1 cells (no stim.) and αCD3ε-treated cells on control DNA probes that lack hairpin stem loop (duplex). d-ICAM-1, dimeric ICAM-1; m-ICAM-1, monomeric ICAM-1. (**C**) Plot showing average hairpin opening underneath the cell-substrate contact. Both monomeric (orange zone) and dimeric ICAM-1 (green zone) ligands were tested. *n* > 15 cells from at least two independent experiments. (**D**) Time-lapse RICM and tension images showing initial landing of αCD3ε-primed cell. (**E**) Displacement plot showing cell tracks of 10 randomly selected αCD3ε-primed cells on the tension probe substrates within 10 min. (**F**) Average LFA-1 tension evolution profile generated from 10 randomly selected αCD3ε-primed cells landing on the 4.7-pN tension probe substrates within the first 10 min. The mean intensities underneath T cells at each time point were normalized to the highest intensity within the sequence. ***P < 0.001; ****P < 0.0001. Scale bars, 5 μm. Probe density = ~1000 molecules/μm^2^.

Previous work using GETS quantified the mechanical tension across the LFA-1 β_2_ tail in Jurkat T lymphoblast and reported a value of ~2 pN. Given that integrins have been reported to bear peak forces >50 pN and equilibrium forces >20 pN (*20*, *29*, *30*), representing values exceeding the dynamic range of GETS (1 to 7 pN), we wondered whether LFA-1 may transmit force magnitudes more akin to other integrin heterodimers. To test this possibility, we used the DNA-based molecular force probes (Fig. 2A) (*31*, *32*). The DNA tension sensor is composed of a DNA stem loop anchored to a glass coverslip through one terminus, while the second end presents a ligand of interest, such as pMHC or ICAM-1 in this work. The hairpin is tagged with a fluorophore and quencher, such that mechanical unfolding of the DNA leads to dequenching of the dye and a ~20-fold enhancement in fluorescence intensity (fig. S5). The unfolding response of these DNA tension sensors is digital; when a cell receptor (e.g., LFA-1 or TCR) applies forces to its ligand with magnitudes exceeding the *F*_1/2_, which is defined as the equilibrium force leading to 50% probability of unfolding, the probe abruptly unfolds and generates a maximal fluorescent response. Most DNA tension sensors are immobilized using biotin-streptavidin because of its tight affinity and rapid association kinetics; however, here, we had to use alternate orthogonal anchoring chemistry because biotin-streptavidin was well suited for pMHC and ICAM-1 conjugation to the oligonucleotide. Hence, we screened different approaches for anchoring DNA sensors to a substrate and identified the copper-free click reaction as an optimal approach to covalently graft the DNA probes onto the glass coverslip.

We treated naïve OT-1 cells with soluble αCD3ε and then immediately plated cells onto glass coverslips presenting dimeric ICAM-1 DNA tension sensors with a *F*_1/2_ of 4.7 pN (probe density = ~1000 molecules/μm^2^; table S2). Cell spreading and LFA-1 tension were quantified using RICM and epifluorescence microscopy, respectively. After ~3 to 4 min of seeding, OT-1 cells engaged and spread on the tension probe substrate and generated force signals underneath the cell contact area (movie S1). Control ICAM-1 DNA duplexes lacking the stem loop did not generate fluorescence signal (Fig. 2B and fig. S6). Also, nonstimulated cells failed to spread, thus confirming that spreading is mediated by primed LFA-1 and the tension signal is driven by mechanical unfolding of the stem loop.

To better define the magnitude of LFA-1 forces, we allowed OT-1 cells to adhere onto hairpin probes with a *F*_1/2_ = 19 pN. Unexpectedly, although the cell adhesion footprint was similar to that of cells plated on *F*_1/2_ = 4.7-pN probes, here, cells generated tension exclusively at the periphery (Fig. 2B). Thus, the highest-magnitude LFA-1 forces are localized to the cell periphery, and this pattern is likely due to the inability of cells to transport the LFA-1/ICAM-1 because the probe was affixed onto the glass coverslip. Further quantification revealed that only ~1.2% of the 19-pN hairpin Probes were opened underneath the cell, whereas ~3% of the 4.7-pN probes were mechanically unfolded by the LFA-1 (Fig. 2C). To rule out the scenario that two LFA-1 receptors simultaneously pull on one tension probe, which would overestimate the forces generated by individual LFA-1, we prepared tension probe substrates (*F*_1/2_ = 4.7 and 19 pN) presenting monomeric ICAM-1 ligand. αCD3ε-stimulated T cells adhered, spread, and migrated on these surfaces and were able to mechanically unfold these probes (~1.9% for 4.7-pN probes and ~0.4% for 19-pN probes; Fig. 2, B and C). These results reveal that the fluorescence signal is a direct consequence of LFA-1 force transmission to the ICAM-1 ligand, with a subpopulation of LFA-1 molecules that transmit forces >19 pN during spreading and migration.

To study LFA-1 force evolution during initial adhesion, spreading, and migration on glass coverslips presenting ICAM-1 tension probes, we acquired time-lapse videos of the tension signal on the dimeric ICAM-1 tension probe substrates for a duration of 10 min and at a frequency of 3 frames/min (Fig. 2D). The data indicated that LFA-1/ICAM-1 forces went through two distinct phases. The first phase was observed upon initial cell adhesion as determined using RICM (within 2 min after cell-surface contact), where 4.7-pN LFA-1 forces were localized to the center of the cell-surface contact junction. Cells rapidly spread during this phase, and the tension signal also rapidly increased. The distribution of the LFA-1 forces was also highly dynamic spreading from the center of the cell-substrate junction to the perimeter. During the second phase, T cell tension reached a “steady state,” and the location of LFA-1 forces was concentrated at the cell periphery. Note that although the tension signal reached a steady state, T cells remained migratory, displaying a polarized phenotype with tension confined to the cell perimeter (Fig. 2, D to F, and fig. S7). Together, primary T cells stimulated with soluble antibodies generate dynamic LFA-1/ICAM-1 forces localized to the cell perimeter. This peripheral localization of LFA-1 forces was similar for the monomeric and dimeric ICAM-1 and was consistent with the accumulation of F-actin at the cell edge in these experiments (fig. S8) as well as recent literature (*33*). PMA and Mg^2+^/EGTA also induced T cell adhesion on the dimeric ICAM-1 tension probe substrates, and these mitogenic stimuli supported force transmission across the LFA-1/ICAM-1 complex with a similar spatial pattern compared to cells stimulated by soluble αCD3ε. However, stabilization of LFA-1/ICAM-1 complex by Mg^2+^ triggered strong T cell adhesion on the dimeric ICAM-1 tension probe substrates with minimal mobility (fig. S7). Cell interacting with the monomeric ICAM-1 tension probe substrates displayed very similar tension signal intensities when comparing the different chemical inducers, with the exception of Mg^2+^/EGTA, which showed relatively lower tension signal compared to the dimeric ICAM-1 (fig. S9).

Actomyosin forces have been proposed to stabilize the LFA-1/ICAM-1 interaction by unclasping the integrin heterodimer (*34*, *35*). Therefore, we plated drug-treated, αCD3ε-primed cells on the 4.7-pN tension probes to measure the effect of cytoskeletal perturbation on LFA-1 forces. Myosin inhibition with blebbistatin (myosin IIa inhibitor) did not induce a notable change LFA-1 tension nor cell spreading area. On the other hand, stabilization of actin turnover by jasplakinolide completely eliminated cell spreading and LFA-1 tension signal. Given that jasplakinolide inhibits actin dynamics by slowing F-actin depolymerization, these results suggest that actin turnover dynamics, rather than myosin contractility, is a primary driver of initial LFA-1–mediated T cell spreading, motility, and tension generation (fig. S10), confirming the recent result reported by Springer and colleagues (*15*).

### Multiplexed DNA tension probes report spatiotemporal dynamics of TCR and LFA-1 forces

**Fig. 3.**
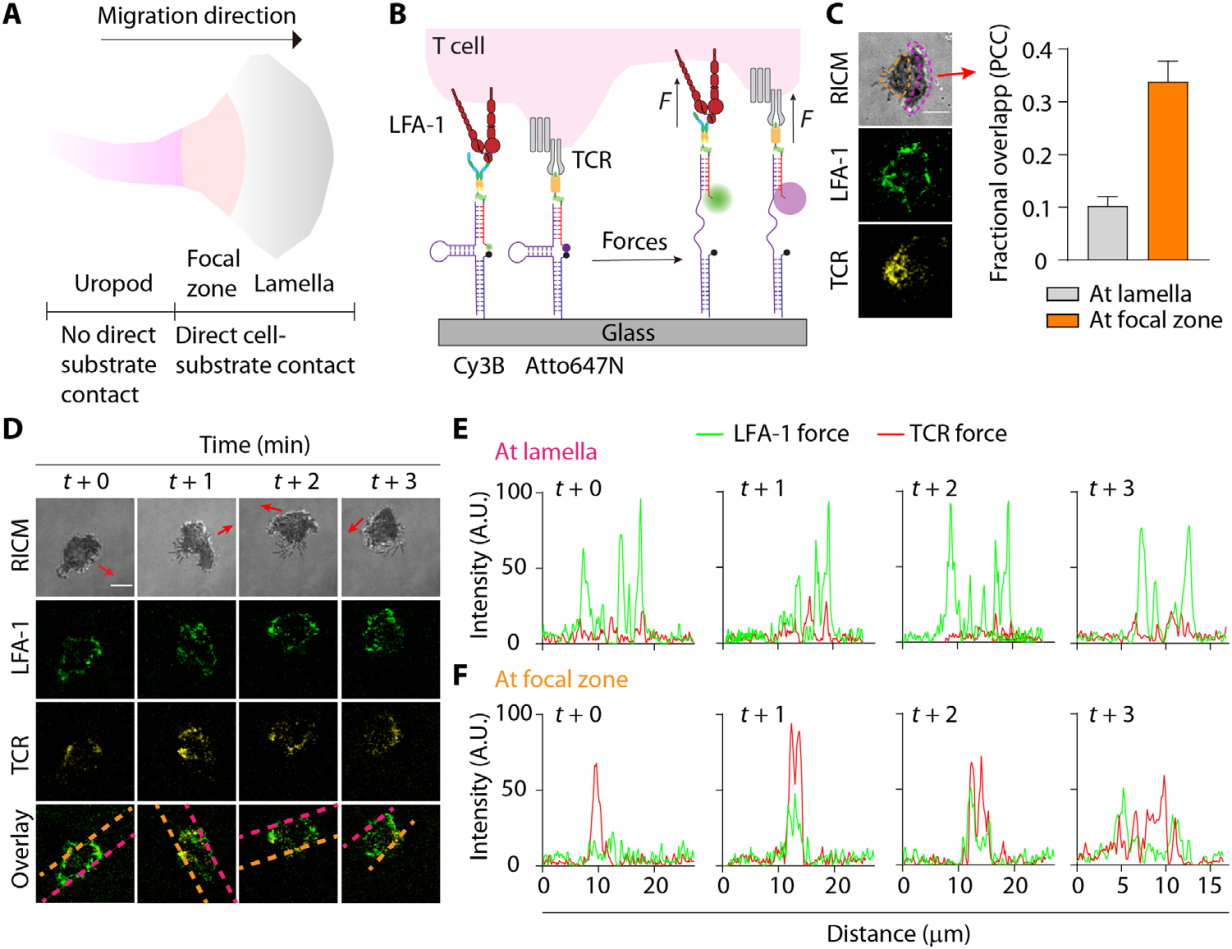
Spectrally encoded, multiplexed extracellular DNA-based TCR and LFA-1 tension probes. (**A**) Morphology of a migrating T cell. (**B**) Design of multiplexed real-time DNA-based tension probes for simultaneous mapping of TCR and LFA-1 forces. (**C**) RICM, LFA-1, and TCR tension images show a migrating OT-1 cell used LFA-1 and TCR to generate forces at spatially distinct regions. Red arrow indicates the direction of migration. Colocalization analysis confirmed the lack of colocalization at the lamella and moderate colocalization at the focal zone. *n* = 15 cells; orange dashed region, focal zone; pink dashed region, lamella. PCC, Pearson’s correlation coefficient. (**D**) Time-lapse images showing cell footprint and spatial segregation of the LFA-1 and TCR forces. Representative RICM and tension maps of the LFA-1 (*F*_1/2_ = 4.7 pN, green), TCR (*F*_1/2_ = 4.7 pN, yellow), and the overlay channel of a single migrating OT-1 cell were shown. For C and D, red arrows indicate the direction of migration. Scale bar, 5 μm. (**E**) Plots display lamella tension signals generated by the LFA-1 and the TCR as a function of time within the line of interest (pink dashed lines) as shown in the overlay channel in (D). (**F**) Plots display focal zone tension signals generated by the LFA-1 and the TCR as a function of time within the line of interest (orange dashed lines) as shown in the overlay channel in (D). Error bar represents means ± SEM. Scale bar, 5 μm. Probe densities = ~1000 molecules/μm^2^. AU, arbitrary units.

Given that the T cell transmits piconewton forces to both the TCR/pMHC and LFA-1/ICAM-1 complexes, we next wondered whether these forces are coordinated spatially and temporally. Traction force microscopy and micropillar arrays have been used to measure how dual presentation of ICAM-1 alongside TCR antibodies may modulate the total traction force (*36*). Nonetheless, these methods are unable to resolve the individual contributions of each ligand-receptor pair. In contrast, DNA-based force probes can be multiplexed to spectrally record individual mechanical contributions of different receptors. To this end, we developed spectrally encoded DNA force probes to simultaneously map forces transmitted across the LFA-1/ICAM-1 and TCR/pMHC bonds in polarized, migrating T cells (Fig. 3A). In particular, we were interested in mapping LFA-1 and TCR forces in the two regions of activated polarized T cells: the lamella and focal zone (*37*, *38*), as the uropod of migrating T cells does not contact the surface.

We generated binary dimeric ICAM-1 and OVA-N4 tension probe surfaces by performing sequential Cu-free click reactions. The ICAM-1 and OVA-N4 probes (*F*_1/2_ = 4.7 pN) were spectrally encoded using Cy3B and Atto647N reporters, respectively (Fig. 3B). As expected, most naïve OT-1 cells seeded on the multiplexed probe surface formed a migratory phenotype and generated tension signal in both channels. We used time-lapse RICM movies to define the lamella and focal zone on the basis of the direction of migration and proximity to cell edge. The LFA-1 forces were primarily found at the lamella (pink dashed region; Fig. 3C), while the TCR/pMHC bonds experienced forces mostly at the focal zone of the migrating cells (orange dashed region; Fig. 3C). The TCR forces moderately colocalized with the LFA-1 forces at the focal zone of migrating T cells, with an average Pearson’s correlation coefficient (PCC) = 0.34 ± 0.04. In contrast, TCR–LFA-1 colocalization was significantly weaker at the lamellipodia (PCC = 0.10 ± 0.02). This result is consistent with our prior observation that TCR forces preferentially localize to the back of migratory T cells coinciding with the focal zone (*39*). To exclude the possibility that this difference was due to artifacts from surface preparation, we labeled the OVA-N4 ligand onto both Cy3B and Atto647N probes so both probes were only capable of reporting TCR tension. Cells did not form a migratory phenotype in the absence of ICAM-1, and we observed strong colocalization of the Cy3B/Atto647N force signal in this case (PCC = 0.72 ± 0.03) (fig. S11).

To investigate the temporal changes of both the TCR and LFA-1 forces, we acquired time-lapse images of migrating cells on the multiplexed tension probes. Both the LFA-1 and TCR forces were highly transient, likely allowing rapid T cell adhesion/deadhesion for antigen sampling (Fig. 3D and movie S2). We performed line scan analysis to investigate the spatiotemporal location of these forces (Fig. 3, E and F). The line scans of LFA-1 and TCR forces confirmed the PCC values and were spatially segregated, where the LFA-1 primarily generated forces at the lamella and the TCR generated forces at the focal zone. This spatial segregation reflects the location of the TCR in the focal zone, while the LFA-1 generated forces at both the lamella and the focal zone. Single-color imaging of LFA-1 force with surface-immobilized pMHC mirrored these results and also showed polarized T cells with forces distributed at the focal zone and the lamellipodia (fig. S12). These results represent the first multiplexed piconewton force map for TCR and LFA-1, highlighting temporally coordinated force at distinct compartments of freshly activated and polarized T cells. The ligand-specific force channels afforded by the multiplexed probes reveal that TCR and LFA-1 cytoskeletal coupling are independent and particularly at the cell leading edge. We also note that these data cannot be obtained by conventional traction force microscopy.

### Mechanical forces through the LFA-1/ICAM-1 bond potentiates TCR-mediated T cell activation and cytokine secretion

**Fig. 4.**
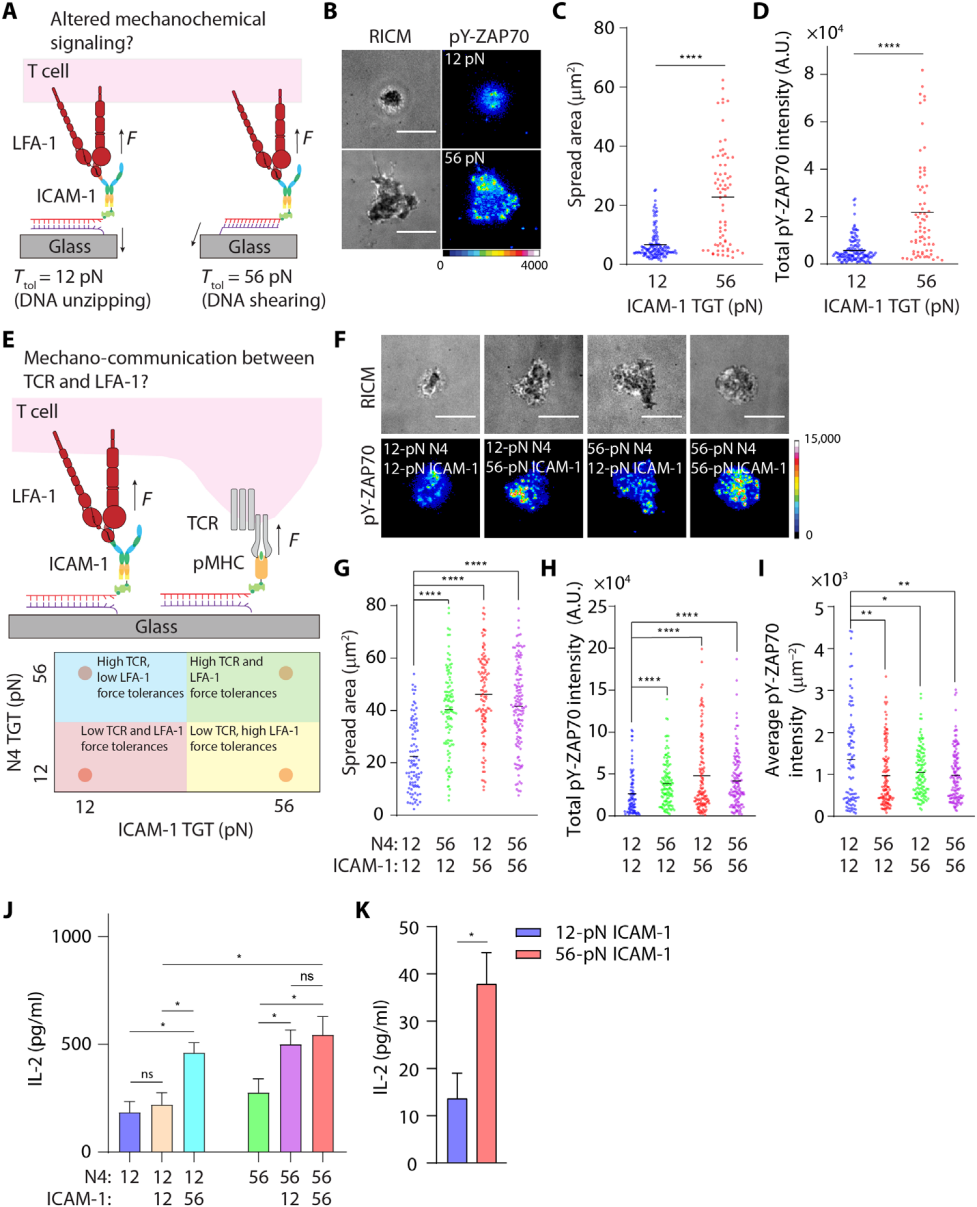
Mechanochemical stabilization of LFA-1/ICAM-1 bonds potentiates TCR-triggered T cell activation and cytokine secretion. (**A**) Schematic showing the design of TGT assays to cap maximal forces that can be transmitted to LFA-1/ICAM-1 bonds. (**B**) RICM and immunofluorescence images showing αCD3ε-primed naïve OT-1 cells spread on ICAM-1 TGT substrates after 1 hour of incubation. Cells were stained with Alexa Fluor 647–pY-ZAP70 antibody. (**C** and **D**) Quantification of spread area and pY-ZAP70 intensity of cells seeded on 12- or 56-pN ICAM-1 TGT substrates. *n* > 50 cells from three independent experiments. (**E**) Schematic showing the design of multifunctional surfaces presenting both ICAM-1 ligands (through the TGT) and immobilized agonist pMHC. (**F**) RICM and immunofluorescence images showing naïve OT-1 cells spread on these multiplexed TGTs after 1 hour of incubation. Cells were stained with Alexa Fluor 647–pY-ZAP70 antibody. (**G** to **I**) Quantification of spread area, total, and average pY-ZAP70 intensity of cells seeded on multiplexed TGT substrates. Dots represent cells pooled from three independent experiments. (**J**) IL-2 production by OT-1 (~1 × 10^5^ cells) after 6 hours of incubation on surfaces presenting N4 TGTs or N4 + ICAM-1 TGTs. (**K**) IL-2 production by αCD3ε-primed OT-1 (~1 × 10^5^ cells) after 24 hours of incubation on surfaces presenting only ICAM-1 TGTs. For (J) and (K), experiments were run using three batches of naïve cells. *P < 0.05; **P < 0.01; ****P < 0.001. Error bar represents means ± SEM. Scale bars, 5 μm. pMHC and ICAM-1 density are estimated to be ~400 to 500 molecules/μm^2^, respectively.

We next examined how the magnitude of LFA-1 tension modulates T cell activation. Some literature suggests that LFA-1 is a co-receptor that augments T cell activation (*40*, *41*), while others indicate that LFA-1 primarily acts as an adhesion molecule (*42*). We hoped to resolve this question and further elucidate the role of mechanics in the context of LFA-1 activation. To gate the LFA-1 mechanical force transmitted to ICAM-1, we used the TGT that is a DNA duplex–anchored ligand that mechanically ruptures at a threshold of tension, or tension tolerance (*T*_tol_) (*20*). In this way, the mechanical rigidity of the ligand can be tuned from 12 to 56 pN, which tunes the maximum (peak) magnitude of mechanical force delivered through this ligand (Fig. 4A). Note that *T*_tol_ of a TGT is orientation independent because it is immobilized onto a substrate with a rotatable bond and the forces applied by the cell receptor dictate the orientation of the TGT (note S1). To test the upper and lower limits in *T*_tol_ values, we plated αCD3ε-primed OT-1 cells on dimeric ICAM-1 TGT surfaces with *T*_tol_ = 12 or 56 pN (Fig. 4B) with probe density = 400 to 500 molecules/μm^2^ (table S2). 56-pN ICAM-1 TGTs triggered more efficient T cell spreading compared to the 12-pN TGTs (Fig. 4C), supporting our earlier results that showed that TCR-stimulated LFA-1 can transmit forces >19 pN on the ICAM-1 ligands. Phosphorylation of ZAP-70 was potentiated by LFA-1 force, as T cells seeded on 56-pN ICAM-1 TGTs displayed increased pY-ZAP70 staining across the entire membrane surface, while T cells on 12-pN TGTs showed minimal pY-ZAP70 activity (Fig. 4D). To confirm that this result is due to differential LFA-1 mechanical signaling, we generated N4 TGTs with *T*_tol_ = 12 and 56 pN and showed also differential pY-ZAP70 levels as we previously reported (*39*). Also, we used a lower-magnification objective to sample whole cells and observed the same difference (fig. S13). These supporting experiments confirm that integrated pY-ZAP70 intensity is a faithful reporter to gauge the strength of T cell signaling. Nonetheless, LFA-1 forces >12 pN applied to dimeric ICAM-1 deliver a signal to enhance TCR-mediated T cell activation.

To quantify the LFA-1–driven TGT probe rupture, we next designed “turn-on” ICAM-1 TGTs where a fluorophore and a quencher are attached to the DNA probes (fig. S14) (*43*, *44*). αCD3ε-primed cells were seeded on turn-on, dimeric ICAM-1 TGT substrates. Fluorescence imaging confirmed that stimulated cells transmit sufficient forces through LFA-1 to rupture the 12-pN ICAM-1 TGTs (fig. S14 and movie S3). The probe rupture pattern was generally disorganized as the turn-on TGT reported an accumulated history of mechanical events and the cells were highly migratory. In contrast, we did not observe any turn-on TGT signal on the 56-pN probe surfaces (fig. S14 and movie S4). Cells on this surface were also highly migratory and showed strong cell-substrate interaction as indicated by the spreading area. As a control, cells seeded on 12-pN N4 TGTs generated strong ring-like turn-on signals, but not the 56-pN TGTs (fig. S15 and movies S5 and S6). The ring size was highly similar to the spread area of cells seeded on 56-pN N4 TGTs, confirming that the TCR generates >12-pN contractile forces to sample antigen. These results indicate that LFA-1 transmits forces >12 pN but <56 pN on the dimeric ICAM-1 ligand and that these receptor forces enhance ZAP-70 signaling. The precise magnitude of LFA-1/ICAM-1 forces is better defined using the reversible DNA-based force probes (Fig. 2) given the time-dependent nature of force-induced duplex rupture (*45*), but nonetheless, here, the TGTs define the role of LFA-1 force magnitude in mediating the strength of TCR-mediated T cell activation.

In light of the potential mechano-communication between TCR and LFA-1, we next developed a multiplexed TGT surface where the N4 and dimeric ICAM-1 ligands were conjugated to TGTs each presenting a unique *T*_tol_ value, resulting in four combinations of ligand and *T*_tol_ (Fig. 4E). Cells seeded on a combinatorial surface with 12-pN N4 TGTs and, 12-pN ICAM-1 TGTs had the lowest adhesion areas and pY-ZAP70 levels. When either ICAM-1 or N4 was anchored to 56-pN TGTs, cells had greater spread area and enhanced total pY-ZAP70 compared to the 12-pN/12-pN surfaces (Fig. 4, F to H). The average pY-ZAP70 density decreased for all groups compared to the 12-pN/12-pN surface likely because of the enhanced cell spreading on the other surfaces (Fig. 4I). Inspired by this observation, we then sought to test how the mechanical communication between TCR and LFA-1 affects T cell functional response. Here, cells were seeded onto the multiplexed TGT surfaces for 6 hours. Supernatants were collected and subjected to quantification of interleukin-2 (IL-2) using enzyme-linked immunosorbent assay. Consistent with the immunostaining result, cells cultured on a combinatorial surface consisting of 12-pN N4 TGTs and, 12-pN ICAM-1 TGTs generated the least IL-2 cytokine compared to other combinatorial surfaces (Fig. 4J). When either ICAM-1 or N4 was anchored to 56-pN TGTs, cells produced an increased amount of IL-2 compared to the 12-pN/12-pN surfaces. Again, presentation of both ligands on 56-pN TGTs did not further increase IL-2 secretion. Unexpectedly, we did not observe any difference in IL-2 production when cells engaged to either single 12-pN or 56-pN N4 TGT, but presentation of ICAM-1 TGT enhanced IL-2 production in all cases except 12-pN/12-pN TGTs (Fig. 4J). As a control, we monitored IL-2 generation of αCD3ε-primed cells seeded onto the ICAM-1 surfaces. We observed little IL-2 generation after 24 hours of culture. Nonetheless, cells seeded on 56-pN ICAM-1 TGT surfaces were able to produce fourfold more IL-2 compared to those on 12-pN ICAM-1 TGT surfaces (Fig. 4K), but to a much lesser extent compared to multiplexed TGT probes. These observations support the idea that the magnitude of LFA-1 force plays a role in strengthening cytokine secretion by activated T cells. Together, both immunostaining and cytokine quantification results show that allowing mechanical forces transmitted through TCR and LFA-1 to exceed 12 pN (56 pN > *F* > 12 pN) triggers an up-regulated level of cell spreading, signal activation, and enhanced cellular response.

### Bidirectional communication between LFA-1 and TCR dictates the strength of T cell activation

**Fig. 5.**
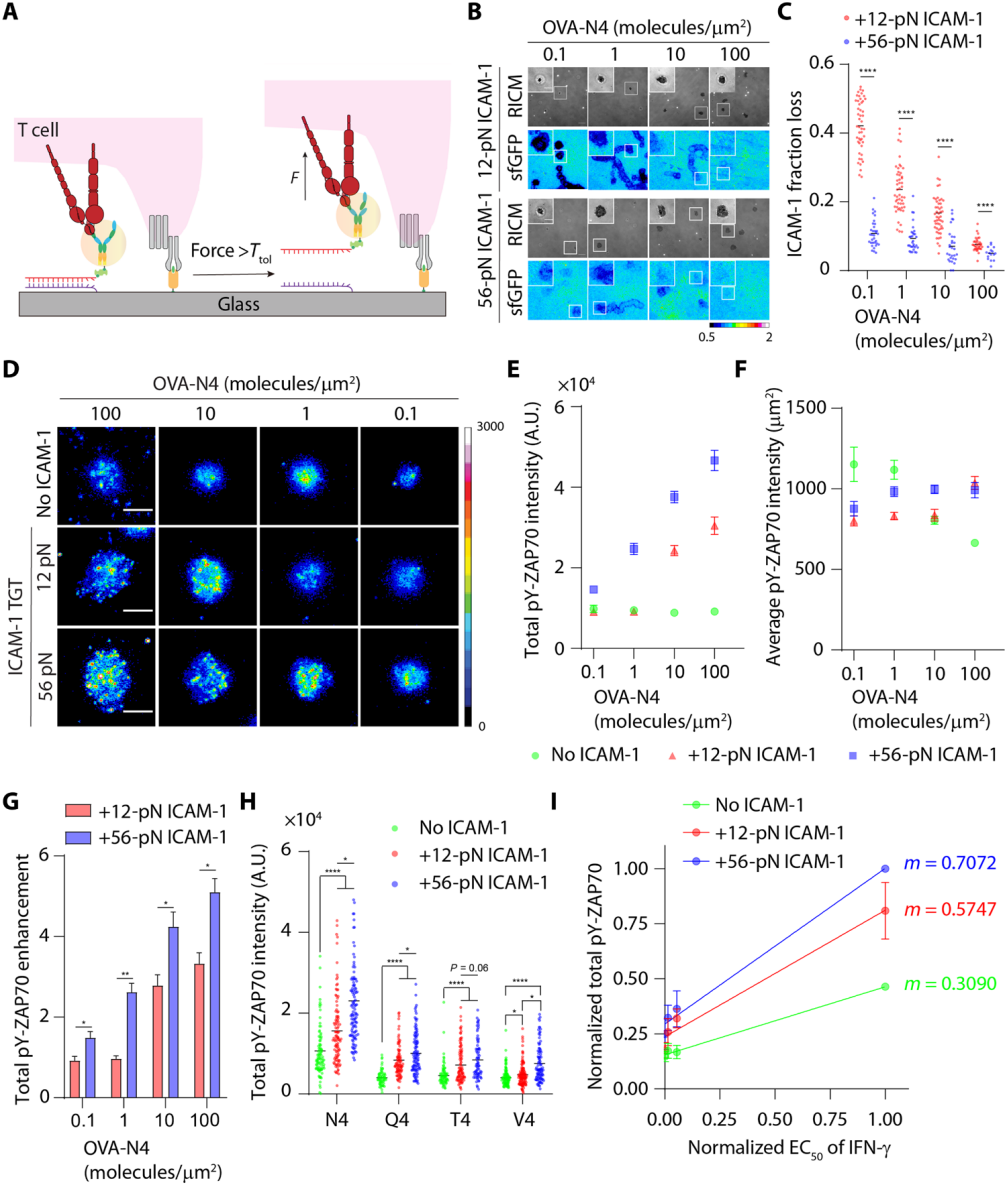
Bidirectional communication between LFA-1 and TCR dictates the strength and specificity of T cell activation. (**A**) Schematic showing the design of stimulatory surfaces presenting both ICAM-1 ligands (through the TGT) and immobilized agonist pMHC. (**B**) Whole-field RICM and fluorescence images (512 × 512 pixels) showing cell spreading and loss of ICAM-1 TGT fluorescence at different antigen densities. Zoom-ins are representative cells. Calibration bar indicates loss of ICAM-1 from the surface. (**C**) Plot quantifying the fraction loss of ICAM-1 at different antigen densities. (**D**) Immunostaining for pY-ZAP70 of cells seeded on surfaces coated with immobilized OVA-N4, immobilized OVA-N4 and 12-pN ICAM1-TGT, or immobilized OVA-N4 and 56-pN ICAM-1 TGT. Antigen densities were varied from ~0.1 to ~100 molecules/μm^2^. (**E** and **F**) Quantification total and average pY-ZAP70 intensity of cells seeded on OVA-N4 only, OVA-N4 and 12-pN ICAM-1 TGT, or OVA-N4 and 56-pN ICAM-1 TGT substrates. (**G**) Plot showing enhancement of pY-ZAP70 intensity of cells seeded on OVA-N4 and 12-pN ICAM-1 TGT or OVA-N4 and 56-pN ICAM-1 TGT substrates compared to those on antigen-only surfaces. (**H**) Plot quantifying the total pY-ZAP70 intensity of cells seeded on surface coated with altered OVA-N4 or altered peptides, OVA-N4 or altered peptides plus 12-pN ICAM-1 TGT, and OVA-N4 or altered peptides plus 56-pN TGT. Antigen density was kept at ~1 molecule/μm^2^. (**I**) Plot of relative functional avidity of N4 or APLs [median effective concentration (EC_50_) data of interferon-γ (IFN-γ) secretion is adapted from (*48*)] against relative total pY-ZAP70–level cells engaging N4 or APLs in the presence (or not) with ICAM-1 TGTs. The slope (*m*) indicates the T cell sensitivity. *P < 0.05, **P < 0.01, ****P < 0.0001. *n* > 50 cells from three independent experiments. Error bar represents means ± SEM. Scale bars, 5 μm. Probe density = 400 to 500 molecules/μm^2^.

We next sought to determine how TCR antigen density (dose) influences ICAM-1 dissociation, which is correlated to the mechanical sampling rate of LFA-1 and also the magnitude of peak force generated by LFA-1. To test this idea, we generated surfaces presenting both the N4 and the ICAM-1. The ICAM-1 was anchored through the TGT to the surface, while the N4 was directly immobilized on the glass surface (Fig. 5A). The N4 density was tuned from ~0.1 to 100 molecules/μm^2^ while keeping the density of ICAM-1 TGT constant (probe density = 400 to 500 molecules/μm^2^; table S2). These densities better mimic expected physiological densities. Unexpectedly, we found that the rupture of ICAM-1 TGTs was highly dependent on the antigen density. At the antigen density of ~0.1 molecules/μm^2^, ~11 ± 0.7% of ICAM-1 molecules on the 56-pN TGT were ruptured from the surfaces in the wake of the migratory T cells, while ~42 ± 1.1% loss of ICAM-1 was observed on the 12-pN TGT. At the highest antigen density tested (~100 molecules/μm^2^), loss of ICAM-1 became less pronounced, dropping to ~5 ± 0.5% for 56-pN TGT and ~7.3 ± 0.3% for 12-pN TGT (Fig. 5, B and C). αCD3ε-primed cells seeded on 56-pN ICAM-1 TGT surfaces without surface-anchored antigen showed a minimal loss of ICAM-1 (1.1 ± 0.07%) (fig. S16). These results show that LFA-1 force exerted by migratory T cells is dependent on antigen density, and TCR triggering by immobilized pMHC enables more ICAM-1 rupture events, indicating a higher frequency of mechanical sampling by LFA-1. Our results also agree with an early observation by Dustin and co-workers (*46*) where the velocity of T cell migration decreased in response to an increase in antigen density on hybrid SLBs containing antigen and ICAM-1. Nonetheless, we showed that most of the LFA-1 exerted peak forces in between 12 and 56 pN, with a subset of LFA-1 that applied a peak force >56 pN, and the TGT rupture became more evident at low antigen densities.

We then asked whether the magnitude of mechanical force through LFA-1 affects early T cell signaling. As expected, cells seeded on N4 surfaces lacking ICAM-1 showed minimal spreading and total pY-ZAP70 levels at all the tested N4 antigen densities from 0.1 to 100 antigen/μm^2^. ICAM-1 tethered through 56-pN TGT showed markedly increased cell spreading and enhanced total pY-ZAP70 levels compared to the 12-pN TGT surfaces across all the antigen densities tested (Fig. 5, D and E, and fig. S17). In general, ICAM-1 tethered through 12-pN TGT resulted in enhancement of both spreading and total pY-ZAP70 levels compared to the surfaces lacking ICAM-1. Note that when the antigen density was low at 0.1 and 1.0 antigen/μm^2^, the ICAM-1 anchored through the 12-pN TGT showed a minimal change in response from the control group lacking ICAM-1 (Fig. 5, D and E). The average pY-ZAP70 intensity of T cells gradually increased in response to surfaces presenting ICAM-1 TGTs and increasing density of N4, while average pY-ZAP70 decreased when T cells encountered surfaces presenting increasing number of N4 without ICAM-1 (Fig. 5F). Overall, these results indicate that, at physiologically relevant densities of antigens, TCR/pMHC engagement alone does not support efficient cytoskeletal remodeling and TCR signaling in the absence of mechanical input through LFA-1/ICAM-1 bonds. This confirms that the T cell fine-tunes its initial antigenic response on the basis of the magnitude of forces transmitted through LFA-1.

We further analyzed the total pY-ZAP70 data by normalizing levels in T cells seeded on surfaces copresenting antigen and ICAM-1 TGTs with different *T*_tol_ to those seeded on antigen-only surfaces (Fig. 5G). The fold enhancement in pY-ZAP70 measures the strength of mechanical sensitization from LFA-1/ICAM-1 bonds to TCR signaling. 56-pN ICAM-1 TGT promoted ~5-fold increase of pY-ZAP70 level at the highest antigen density tested (100 antigens/μm^2^). Similarly, ZAP70 signaling was also enhanced with the 12-pN ICAM-1 TGT, but less potently, showing a ~3.3-fold signal enhancement at an antigen density of 100 molecules/μm^2^. Unexpectedly, the signal gain from LFA-1/ICAM-1 interaction was completely abolished in the 12-pN TGT at 0.1 antigen/μm^2^. Collectively, these data indicate that mechanically robust LFA-1/ICAM-1 bonds (i.e., ICAM-1 tethered to 56-pN TGT) act as amplifiers to strengthen early T cell signaling by enhancing and stabilizing cell spreading at the cell-substrate interface.

### Mechanical force transmitted through LFA-1/ICAM-1 bond enhances antigen discrimination

Because LFA-1 mechanical force amplifies early T cell signaling by enhancing T cell sensitivity to its cognate antigen, we hypothesized that this force may also boost the discriminative power (i.e., selectivity) of the TCR. Here, we used a panel of altered peptide ligands (APLs) of the parental N4 pMHC, and we tested whether the high-tension LFA-1/ICAM-1 bond augmented antigen discrimination. We choose three APLs that differ in single–amino acid substitution at the fourth position: Q4 (SIIQFEKL), T4 (SIITFEKL), and V4 (SIIVFEKL). These APLs represent a ~24-fold range of two-dimensional (2D) binding affinity (*47*) and a ~680-fold range in functional avidity (*48*) in the order of N4 > Q4 > T4 > V4.

We prepared stimulatory surfaces as shown in Fig. 5A and held the density of N4 or APLs at ~1 molecule/μm^2^. We found that the presence of LFA-1/ICAM-1 and the mechanical strength of this bond were critical in boosting early T cell activation (pY-ZAP70) (Fig. 5H and fig. S18) for the cognate antigen as well as the APLs tested. To quantify the specificity of the T cell response to the APLs, we generated a plot where the reported functional avidity (median effective concentration of interferon-γ response) of each antigen (*48*) was plotted on the *x* axis and the pY-ZAP70 response measured here was plotted on the *y* axis. The slope of the 56-pN group was greater than that of the 12 and the no-ICAM substrates. Given that the slope is an indicator of the specificity of the response, this experiment shows that the mechanical strength of the LFA-1/ICAM-1 further enhances the discriminatory power of TCR toward antigens with different binding affinities. This conclusion is consistent with prior reports that showed TCR-pMHC forces in the 10- to 20-pN range boost the specificity of the TCR in distinguishing between APLs (*18*, *39*, *49*). We note that the relative pY-ZAP70 values for the 12-pN ICAM-1 surfaces were greater in the experimental sets in Fig. 5 (H and I) compared to Fig. 5 (D to F). This discrepancy could be due to differences in the intrinsic T cell reactivity, where results presented in Fig. 5 (H and I) were generated using naïve cells isolated from homozygous OT-1 mice.

## DISCUSSION

TCR-mediated activation of LFA-1 leads to modulation of its affinity and avidity and is important for initiating the “stop signal” and allowing T cells to fully engage APCs, which is a hallmark of T cell activation (*50*, *51*). However, the mechanism of LFA-1 “activation” in the context of mechanical force transmission and its ability to sense biophysical cues to support TCR signaling are less explored. Using SLBs and ligand-coated glass coverslips as stimulatory surfaces, we provide multiple lines of evidence to show that robust mechanical interaction of LFA-1/ICAM-1 is needed to support T cell adhesion and signaling especially at relatively low antigen densities. Furthermore, we show that the magnitude of this force is important and tunes T cell signaling and further enhances antigen discrimination.

First, we demonstrate that TCR activation by αCD3ε cross-linking is insufficient to trigger LFA-1/ICAM-1 anchorage and spreading on fluid membranes but readily drives LFA-1 bond formation with immobile ICAM-1 (Fig. 1). In addition, a uniform monolayer of pMHC presented on fluid SLBs at superphysiological density does not provide stable anchoring for the TCR in the absence of LFA-1/ICAM-1 interactions. This suggests that force orientation matters as the fluid SLB offers mechanical resistance in the perpendicular direction but not in the lateral direction; lateral resistive forces on fluid SLB are approximately in femtonewtons (*52*), while phospholipid extraction from the bilayer requires tens of piconewtons (*53*, *54*). The TCR/pMHC interaction is dampened on fluid DOPC membranes (fig. S4) in the absence of ICAM-1, but copresentation of ICAM-1 rescues the DOPC membranes and renders it as highly stimulatory. When these ligands were presented onto a DPPC bilayer that has limited lateral mobility, T cells also formed stable adhesion and initiated early signaling albeit with weaker responses compared to cells stimulated on a DOPC bilayer (fig. S4). These results underscore the importance of LFA-1/ICAM-1 mechanics in maintaining sustained and durable T cell signaling and stable APC contacts, supporting a force-dependent signaling mechanism through LFA-1/ICAM-1. While the current study did not explore how differential mobility of pMHC and ICAM-1 affects T cell signaling, such possibility could potentially be achieved using a hybrid bilayer system. In this hypothetical setup, ICAM-1 is immobilized on a hexagonal gold nanoparticle array with defined interparticle spacing and pMHC is embedded onto a fluid bilayer (*55*). Further enhancing physiological relevance, the SLB could be deposited onto soft polydimethylsiloxane substrates mimicking the physiological relevant stiffnesses of APCs (*56*). These proposed experimental systems will enable a systemic investigation on how the immobilized pool of ICAM-1 alters the ability of naïve cells to discriminate antigens, T cell adhesion, TCR signaling, and actin dynamics.

The second line of experiments that support the role of LFA-1/ICAM-1 forces in TCR signaling comes from spatial and temporal mapping of LFA-1 forces of primary T cells transmitted to anchored ICAM-1 by using reversible DNA-based tension probes. We found that TCR-triggered LFA-1 transmits forces to the ICAM-1 with magnitudes that generally exceed 4.7 pN, and a subset of receptors, possibly high-affinity LFA-1/ICAM-1 bonds, is able to generate forces >19 pN. Our results show LFA-1 forces one order of magnitude greater than the ~2-pN forces transmitted across the β_2_ tail of LFA-1 reported by Springer and colleagues (*15*). This is likely due to several differences between our measurements. The GETS sensor reports the ensemble average tension, while the DNA probes are digital and hence the fluorescence intensity reports that absolute number of mechanical events exceeding the *F*_1/2_. The GETS probes will average and conceal subpopulations of receptors that have greater magnitude of tension. Moreover, measurements in primary T cells are fundamentally different from those in immortalized cell lines. Another difference is that the GETS probe measures intracellular forces within the β tail of the integrin, whereas we are directly measuring the ligand-receptor forces. Sheetz and others (*57*) showed that mechanical unfolding of talin leads to vinculin binding, and the response was enhanced when the externally applied force exceeded ~12 pN compared to talin-vinculin binding under 2 pN. More recent force spectroscopy work by Yan, Sheetz, Goult, and others (*58*, *59*) further underscores this point and shows that different domains of talin will unfold at increasing thresholds of tension that range from 5 to 15 pN. Molecular force measurements using an improved GETS probe by Grashoff and others (*60*, *61*) showed that talin molecules are exposed to *F* > 7 pN in fibroblasts during adhesion. Together, this body of growing evidence is consistent with our measurements of LFA-1 force that exceeds 5 pN and reflects coupling of multiple motor proteins to LFA-1 through the cytoskeleton.

Our force measurements are consistent with the biomembrane force probe results that showed that the LFA-1/ICAM-1 bond displays catch bond behavior at 10 pN, meaning that the bond lifetime is enhanced when the complex experiences 10 pN of tension (*62*). We also note that both monomeric ICAM-1 and dimeric ICAM-1 can support efficient T cell adhesion and force generation with maximal forces >19 pN when the LFA-1 activation is triggered by an inside-out signal (e.g., αCD3ε and PMA). When LFA-1 is activated by an outside-in signal (e.g., Mg^2+/^EGTA), only dimeric ICAM-1 can support T cell adhesion and LFA-1 force generation, highlighting that LFA-1 microclustering/avidity modulation is needed for LFA-1–dependent T cell adhesion.

Under physiological conditions, surface receptors on T cells work cooperatively to form durable T cell–APC contact that elicits precise regulation of T cell response (*63*). Prior work to study the mechanical cross-talk between TCR and LFA-1 includes the use of micropatterned substrates by Kam and others (*36*), where they confirmed that surface-displayed ICAM-1 enhances the total traction forces applied by T cells. Here, we developed multiplex tension probes to dissect force generation and mechanochemical feedback between these two receptors. As shown in this work, the multiplexed tension sensors resolve molecular forces transmitted by each receptor with high spatial and temporal resolution (Fig. 3). We showed that both the TCR and the LFA-1 apply forces to their cognate ligands within distinct compartments in migratory T cells, and these forces modulate initial T cell activation. Given that the zones of LFA-1 and TCR tension are spatially segregated and are also known to be biochemically distinct, this suggests that each pair of receptors possibly engages the same actin network in different ways to mediate control over the magnitude and duration of mechanical force transmission (*64*). Previous work demonstrated that the cytoplasmic tail of LFA-1 is associated with a specific set of adaptor proteins such as talin, ezrin, vinculin, and kindlin and Wiskott–Aldrich Syndrome protein (WASp) (*65*, *66*), while TCR may be linked to actin with a partially overlapping set of adaptor proteins such as non-catalytic region of tyrosine kinase (Nck), VAV, and WASp (*67*). While we did not investigate these adaptor proteins in the present work, the tools described here provide an approach toward resolving the role of these adaptor proteins in tuning the spatial and temporal aspects of force transmission of LFA-1.

Using pY-ZAP70 as a proxy of the strength of T cell activation, we demonstrate that T cells could not be effectively activated at low pMHC densities without co-receptor engagement (Fig. 5B). We show that T cell response was further tuned by the mechanical strength of LFA-1/ICAM-1 bond, where T cell activation was increased at low pMHC densities when ICAM-1 was tethered to 56-pN TGTs. Thus, signaling augmentation through LFA-1/ICAM-1 appears to sensitize the antigenic signaling. A subset of ICAM-1 on 56-pN TGTs was ruptured only when T cells encounter surface antigens (indicated by the loss of ICAM-1 fluorescence showed in Fig. 5B) but not the activating αCD3ε antibody in solution (fig. S16). This suggests that the magnitude of LFA-1 tension is positively regulated by confined TCR-pMHC interactions.

**Fig. 6.**
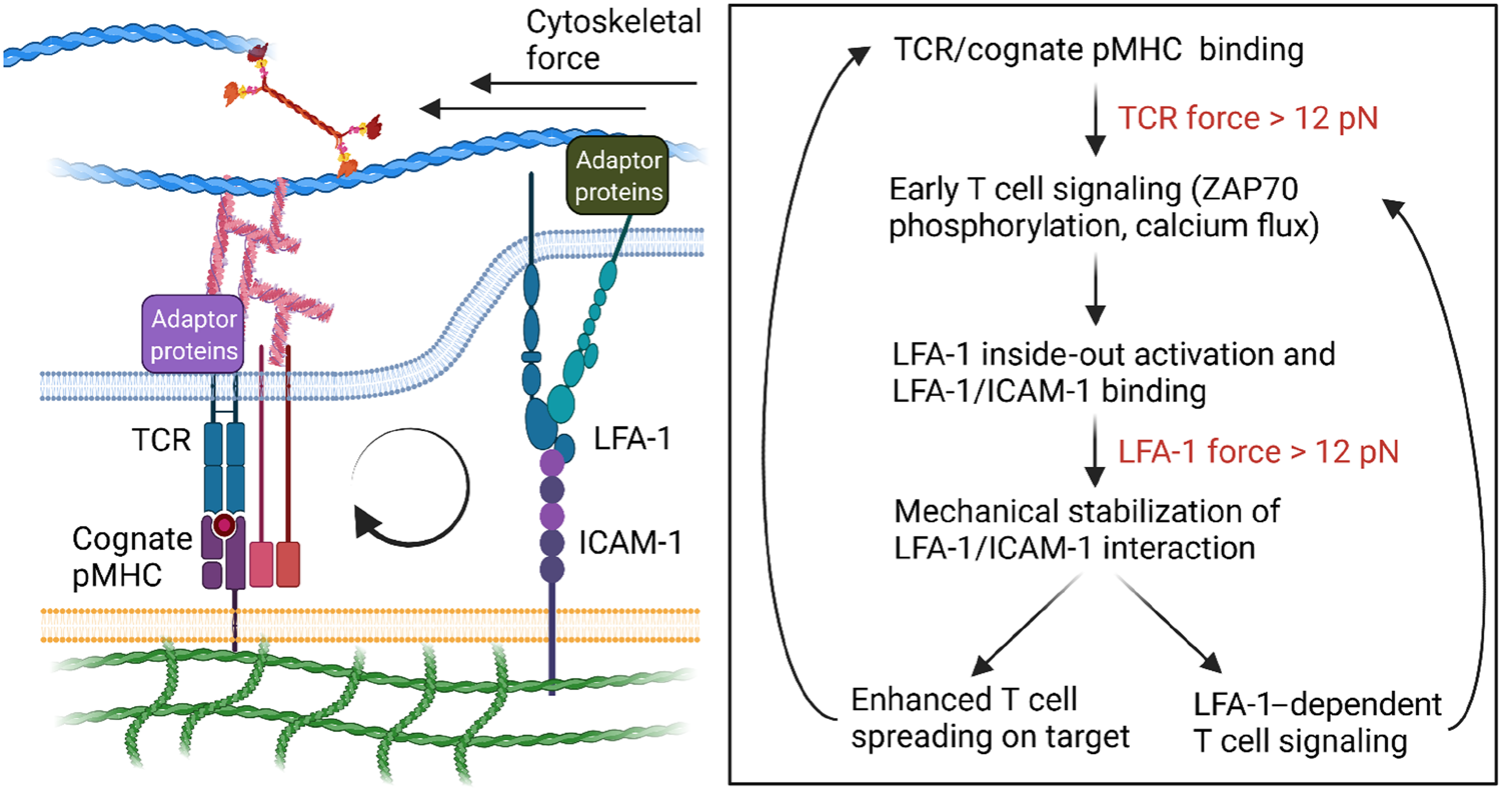
Proposed mechano-communication mechanism in antigen discrimination. When a T cell encounters a cognate antigen presented on an APC, initial force-dependent TCR-pMHC interactions drive productive downstream TCR signaling, LFA-1 “inside-out” activation, and cytoskeletal remodeling. Primed LFA-1 recognizes ICAM-1 on the APC, and this leads to enhanced cell adhesion and spreading. TCR signaling is further enhanced by mechanically robust LFA-1/ICAM interactions. First, mechanically stabilized LFA-1/ICAM-1 binding enables TCR to sample more antigens. Second, LFA-1/ICAM-1 bonds may drive a mechanochemical feedback loop to tune the force transmitted through TCR and itself may provide additional chemical signals to augment T cell activation. Weak peptide ligands do not mechanically benefit from the TCR/LFA-1 cross-talk because these APLs fail to form sustained mechanical bonds with TCR when compared to the cognate antigen.

Our results paint a more complete picture of the force-dependent mechanism of T cell activation (Fig. 6). Previously, it was demonstrated that the TCR scans antigens through a mechanical stringency test, where cells engaging a cognate ligand selectively displays enhanced TCR-dependent phosphorylation, presumably due to the prolonged bond lifetime under the influence of mechanical load (*18*, *39*, *49*). Here, we demonstrated that mechanically stabilized LFA-1/ICAM-1 bonds (*F* > 12 pN) allow T cells to spread and signal more effectively at the cell-substrate interface. The mechanically stabilized bonds allow T cell to sample more antigens on the substrate by increasing TCR occupancy or improving the likelihood of kinetic proofreading/serial engagement of TCR toward the same, cognate antigen (*68*). It would be interesting to further validate this point by using a nonimmune-related adhesion molecule that engages actin cytoskeleton but not eliciting immune cell signaling (*62*). It is also possible that mechanically robust LFA-1/ICAM-1 interactions could amplify TCR signaling by providing feedback via biochemical and/or mechanical pathways. For instance, TCR signaling kinases (e.g., Lck and ZAP-70) coimmunoprecipitated with LFA-1 even in unstimulated T cells and may have a major role in controlling T cell migration and adhesion strengthening (*69*). Also, TCR and LFA-1 have been shown to engage to a common upstream actin network, where the cytoskeletal tension imparted on the respective TCR/pMHC or LFA-1/ICAM-1 bonds could be dependent on each other (*36*).

There are limitations and caveats in using these probes to map receptor-specific molecular forces. For instance, the reported DNA-based tension probes use a tetravalent streptavidin for conjugating ligands where the distance between two biotin binding pockets is approximately 2 nm (*70*). Accordingly, it is possible that multiple ligands could be tethered to a single DNA probe, resulting in overestimation of forces due to simultaneous pulling from multiple receptors. This possibility is unlikely due to the bulkiness of the pMHC/ICAM-1 ligands (with dimensions greater than that of streptavidin). Also, although the tension probes enable concurrent mapping of the spatiotemporal changes of TCR/pMHC and LFA-1/ICAM-1 forces on artificial glass substrates, it is likely that the spatial patterns observed in Figs. 2 and 3 do not represent force transmission by these receptors at the immunological synapse formed by a T cell and an APC in vivo. In addition, we note that the magnitude and lifetime of TCR or LFA-1 tension is likely dependent on T cell subtypes, antigen, and the activation state of the T cell. To better recapitulate this scenario, tension probes presenting ICAM-1 and pMHC could be anchored on fluid SLB or live APCs with appropriate chemistries (e.g., cholesterol insertion into membrane) and subsequently perform fluorescence lifetime imaging microscopy to monitor tension probe opening (*71*). Last, our measurement is unable to capture the rate of pulling and the maximum displacement amplitudes generated by LFA-1 or TCR, and methods to image forces of these receptor at the single-molecule level may be helpful to elucidate their kinetic force profiles (i.e., loading rates and 2D binding) (*72*).

To conclude, our results demonstrate that LFA-1 alone can transmit piconewton forces to ICAM-1 ligand and the force transmission could be further enhanced when a cognate antigen is immobilized on the same plane. Also, we clearly show that forces transmitted by TCR and LFA-1 are compartmentalized and their associated signaling assemblies, although spatially distinct, can converge mechanical signals from integrins and TCRs to optimize the extent of T cell activation. Our results echo the recent studies by Kam and others (*36*) and Burkhardt (*73*) where they showed that TCR and LFA-1 mechanically interact with a common upstream cytoskeletal network and the signaling cross-talk between these two receptors is optimal when engaging their respective ligands in cis. With these molecular tension–sensing platforms in hand, it would be interesting to test how different integrin adaptors recruited by low- and high-tension LFA-1/ICAM-1 bonds communicate with different TCR assemblies. We also anticipate using this platform technology to identify intracellular proteins that serve as intermediates to control the mechanically driven signaling between the TCR/LFA-1.

## MATERIALS AND METHODS

### Reagents

Reagents, unless otherwise specified, were purchased from Sigma-Aldrich (St. Louis, MO) and used as received. All solvents were of analytical grade and purified as needed. No. 1.5H coverslips (#10812) and sticky-Slide VI 0.4 (#80608) were purchased from ibidi (Fitchburg, WI). Cy3B–*N*-hydroxysuccinimide (NHS) ester (#PA63101) was purchased from GE Healthcare Life Sciences (Pittsburgh, PA). Azide-PEG_4_-NHS ester (#AZ-103) was obtained from Click Chemistry Tools (Scottsdale, AZ). RPMI 1640 and Dulbecco’s modified Eagle’s medium (DMEM) (#10-103-CV), heat-inactivated fetal bovine serum (FBS) (#35-015-CV), penicillin-streptomycin solution (#30-234-CI), and gentamicin sulfate solution (#30-005-CR) were purchased from Corning Mediatech (Corning, NY). DOPC (#850375C) and DPPC were purchased from Avanti Polar Lipids Inc. (Alabaster, AL). Texas Red–1,2-dihexanoyl-*sn*-glycero-3-phosphoethanolamine (TR-DHPE) (#23301) was purchased from AAT Bioquest (Sunnyvale, CA). Oligonucleotides (table S3) were obtained from Integrative DNA Technologies (Coralville, IA) and were purified either by reverse-phase high-performance liquid chromatography (HPLC) or standard desalting. Transfection grade linear polyethylenimine (#23966-1) was purchased from Polysciences Inc. (Warrington, PA). All buffer solutions were made with Milli-Q water (18.2 megohms cm^−1^) and passed through a 0.2-μm filtration system.

### Antibodies and biotinylated pMHC

Purified anti-mouse CD3ε (clone 145-2C11, #100302) was purchased from BioLegend (San Diego, CA). Alexa Fluor 647 Mouse Anti-ZAP70 (PY319)/Syk (PY352) (#557817) was obtained from BD Biosciences. H-2K^b^ monomer with the agonist OVA peptide SIINFEKL (OVA-N4) was obtained from the National Institutes of Health (NIH) Tetramer Core Facility (Emory University, Atlanta, GA, USA). The OVA-N4 peptide was synthesized on a Prelude peptide synthesizer (Gyro Protein Technologies) using fluorenylmethyloxycarbonyl chemistry and resuspended in Milli-Q water to 3 mM and sterile-filtered through a 0.22-mm syringe filter.

### General experimental procedures

Concentrations of purified oligonucleotide conjugates and the ICAM-1 proteins were determined on a NanoDrop 2000 UV-Vis Spectrophotometer (Thermo Fisher Scientific). Matrix-assisted laser desorption/ionization–time-of-flight (MALDI-TOF) mass spectrometry was performed on a high-performance MALDI-TOF mass spectrometer (Voyager STR). Matrix for DNA analysis was prepared by freshly dissolving excess 3-hydroxypicolinic acid in the solvent [50% MeCN/H_2_O, 1% trifluoroacetic acid, 10% of ammonium citrate (50 mg/ml)].

### Synthesis of dye-labeled DNA strand

The strand is prepared following a reported protocol (*74*). Briefly, a mixture of A21B (10 nmol) and excess Cy3B-NHS ester or Atto647N-NHS ester (50 μg) in 0.1 M sodium bicarbonate solution was allowed to react at RT overnight. The mixture was then subjected to P2 gel filtration to remove salts, organic solvent, and unreacted reactants and was further purified by reverse phase HPLC (solvent A: 0.1 M Triethylamine acetate, solvent B: acetonitrile; initial condition was 10% B with a gradient of 1%/min, flow rate: 1 ml/min). The product was characterized by MALDI-TOF mass spectrometry.

### Harvest and purification of OT-1 cells

OT-1 TCR transgenic mice were housed and bred in the Division of Animal Resources Facility at Emory University in accordance with the Institutional Animal Care and Use Committee. OT-1 T cells express the CD8 co-receptor and specifically recognize chicken OVA epitope 257-264 (SIINFEKL) in the context of the MHC allele H-2K^b^. For most experiments, naïve CD8^+^ T cells were enriched from the spleen of heterozygous OT-1 mice [except cells used in Fig. 5 (H and I) that were obtained from homozygous OT-1 animals] using magnetic-activated cell sorting according to the manufacturer’s instructions provided with the CD8a^+^ T cell Isolation Kit (Miltenyi Biotec, Germany). Briefly, a single-cell suspension of splenocytes was obtained and incubated with biotinylated antibodies specific for unwanted splenic cell populations. These populations were separated from the OT-1 T cells following incubation with antibiotin magnetic beads and enrichment on a magnetic column. Purified T cells were washed and resuspended in Hanks’ balanced salt solution (HBSS) and kept on ice before experiment.

### Plasmids

The DNA sequence of the monomeric ICAM-1 or dimeric ICAM-1 was ligated to the LentiORF pLEX-MCS vector (Thermo Fisher Scientific) with the help from the Emory Integrated Genomics Core (see amino acid sequences in table S1).

### Lentivirus production and generation of cell lines stably expressing soluble, biotinylated-recombinant ICAM-1s

Human embryonic kidney (HEK) 293FT cells for lentivirus production were maintained in complete DMEM [10% FBS, penicillin G (100 IU/ml), and streptomycin (100 μg/ml)] at 37°C with 5% CO_2_. Lentivirus particles were produced HEK293FT cells in 1× T225 flasks by cotransfection of the pLEX transfer plasmid with the second-generation packaging plasmids pMD2.G and psPAX2 (a gift from D. Trono, Addgene plasmids #12259 and #12260) using linear polyethylenimine (molecular weight = 25,000). The particles were harvested from the supernatant 60 to 72 hours after transfection, filtered, and concentrated into ~250 μl in D20 media (DMEM + 20% FBS) by ultracentrifugation and stored in −80°C before use.

For lentiviral transduction, ~20,000 TB-15 cells (a variant of HEK293T cells stably expressing BirA biotinylating enzyme, a gift from J. Altman and R. Willis from the NIH Tetramer Core Facility at Emory University) were seeded onto a 96-well cell culture plate. On the next day, ~50 μl of concentrated lentivirus particles was added to the cells. After 6 hours of infection, the media were exchanged to complete DMEM. Transduced cells were expanded to appropriate density before adaptation to suspension culture. We used FreeStyle 293 expression media supplemented with 100 μM d-biotin for direct adaptation in a shaking incubator (8% CO_2_, shaking speed = 120 rpm). Soluble ICAM-1 molecules were collected from the supernatant every 3 days or when the cell density reached ~1,000,000 cells/ml.

### Purification of biotinylated ICAM-1s from suspension culture

The harvested supernatant was centrifuged for 5 min at 1200 rpm to pallet the cells. The supernatant was then carefully removed and filtered using a 0.2-μm filter to remove any remaining cells in the solution. To the filtered solution, 50 mM tris (pH 7.5), 500 mM NaCl, and 10 mM imidazole were added, and the resulting solution was directly added to nickel– nitrilotriacetic acid agarose bead (200-ml supernatant per 1-ml bead). The solution was incubated on a rotary platform overnight at 4°C. On the next day, the bead was packed to a plastic polypropylene column and washed with 10× column volume of 50 mM tris (pH 7.5), 500 mM NaCl, and 10 mM imidazole. Last, the protein was eluted with 50 mM tris (pH 7.5), 500 mM NaCl, and 1 M imidazole. The eluted solution was concentrated using an Amicon Ultra-15 centrifugal filter and buffer-exchanged into 1× phosphate-buffered saline (PBS). The concentrations of the ICAM-1s were adjusted to 1 mg/ml and stored at −80°C before use.

### Fluorescence microscopy

The microscope was a Nikon Eclipse Ti driven by the Elements software package. The microscope features an Evolve electron-multiplying charge-coupled device (Photometrics), an Intensilight epifluorescence source (Nikon), a CFI Apo 100× (numerical aperture 1.49) objective (Nikon) and a total internal reflection fluorescence launcher with three laser lines: 488 (10 mW), 561 (50 mW), and 638 nm (20 mW). This microscope also includes the Nikon Perfect Focus System, an interferometry-based focus lock that allowed the capture of multipoint and time-lapse images without loss of focus. In all the reported experiments, we used the following Chroma filter cubes: TRITC, Cy5, and RICM.

### Preparation of small unilamellar vesicle

Small unilamellar vesicle (SUV) was prepared according to reported protocols (*75*, *76*). Briefly, lipids with desired composition were mixed in a round-bottom flask. The lipid mixture was dried using a rotary evaporator to remove the chloroform. The lipids were further dried under a steam of compressed N_2_ and then hydrated with 2 ml of Milli-Q water to a concentration of 2 mg/ml. Three cycles of freeze-thaw were performed to completely dissolve the lipids. The resulting lipids were then repeatedly extruded through a 80-nm polycarbonate filter until the solution became clear (~10 times) and stored at 4°C. The extruded SUVs are stable for 4 to 6 weeks.

### SLB formation

The wells in optically transparent 96-well plates were cleaned with 200 μl of 200 proof ethanol for 5 min at RT and washed thrice with Milli-Q water. Then, the wells were further cleaned with 200 μl of 1% (w/v) KOH for 10 min at RT, washed thrice with Milli-Q water, and completely dried. The plate was treated with plasma for 3 min. After the plasma treatment, the vesicles (0.5 mg/ml) were added to a cleaned 96-well plate and allowed to spread for 30 min at 50°C. Unbounded vesicles were removed with three washes of 1× PBS. SLBs were subsequently blocked with 0.1% bovine serum albumin (BSA) in 1× PBS for 30 min, and the substrates were washed thrice with 1× PBS. Then, streptavidin (10 μg/ml) was added and incubated with the substrates for 45 min. Wells were washed thrice with 1× PBS. Subsequently, biotinylated ligand(s) (5 μg/ml) were added and incubated for 45 min. Wells were washed with 1× PBS and buffer-exchanged with HBSS before imaging.

### Quantitative fluorescence microscopy

Surface density of tension probes and ICAM-1 was measured using a quantitative fluorescence microscopy technique developed by Groves and others (*77*). Briefly, SUVs containing 0.1 mole percent (mol %) TR-DHPE (Invitrogen Corp.) and 99.9 mol % DOPC were mixed to generate vesicle mixtures with TR-DHPE ranged from 0 to 0.1 mol %. These solutions were diluted to 0.5 mg/ml and added to a cleaned 96-well plate to establish a lipid calibration curve. We estimate the molecular density of DOPC lipid molecules using the experimental footprint (~0.72 nm^2^); therefore, a maximum molecular density of ~2.78 × 10^6^ could be packed in 1-μm^2^ surface. The bilayer TR fluorescence was plotted as a function of the molecular density of TR-DHPE. A linear regression was fitted through the origin, and this slope was designated as *I*_bilayer(lipid)_.

To use the fluorescent lipid bilayer calibration curve, a scaling factor (*F* factor) is introduced to account for the difference in brightness between the samples and TR-DHPE. We prepared varying concentrations (50 to 200 nM) of samples and the TR-DHPE liposome and compared their intensities at the same concentration to obtain the *F* factor. The *F* factor is defined as

*F* = *I*_solution(sample)_/*I*_solution(TR-DHPE)_

where *I*_solution(sample)_ and *I*_solution(TR-DHPE)_ represent the intensity of the sample and the TR-DHPE, respectively, in solution at identical concentrations.

The *I*_solution(sample)_ and *I*_solution(TR-DHPE)_ can be estimated by establishing solution-based calibration plots, where the fluorescence intensity of each sample was plotted as a function of its concentration and fitted to a linear regression to obtain the slope (*I*_solution_). The slope of sample *I*_solution(sample)_ was directly compared to the *I*_solution(TR-DHPE)_ to yield a *F* factor. The *F* factor was subsequently used to infer the molecular density of the sample from the SLB calibration.

### Preparation of DNA hairpin-based molecular tension probes on glass surfaces

No. 1.5H glass coverslips (ibidi) were sequentially sonicated in Milli-Q water (18.2 megohms cm^−1^) and 200 proof ethanol, 10 min each. The glass slides were rinsed copiously with Milli-Q water and immersed in freshly prepared piranha solution (3:1 sulfuric acid:H_2_O_2_) for 30 min (CAUTION: Piranha is highly reactive and explosive on contact with organics!). The cleaned substrates were then rinsed with Milli-Q water in a 200-ml beaker at least six times and further washed with ethanol thrice. Slides were then transferred to a 200-ml beaker containing 3% 3-aminopropyltriethoxysilane (APTES) in ethanol for 1 hour, washed with ethanol thrice, and dried with N_2_. The slides were then mounted onto a six-channel microfluidic cells (Sticky-Slide VI 0.4, ibidi). To each channel, ~50 μl of NHS-PEG_4_-azide (10 mg/ml) (Click Chemistry Tools) in 0.1 M NaHCO_3_ (pH 9) was added and incubated for 1 hour. The channels were washed with 1 ml of Milli-Q water thrice, and then the remaining water in the channels was removed by pipetting. The surfaces were further blocked with 0.1% BSA in 1× PBS for 30 min and washed thrice with 1× PBS, and ~50 μl of solution was kept inside the channel to prevent drying. Subsequently, the hairpin tension probes were assembled in 1 M NaCl by mixing the Cy3B-labeled A21B strand (220 nM), quencher strand (220 nM), and hairpin strand (200 nM) in the ratio of 1.1:1.1:1. The mixture was heat-annealed at 95°C for 5 min and cooled down to 25°C over a 30-min time window. The assembled probe (~50 μl) was added to the channels (total volume = ~100 μl) and incubated overnight at RT. This strategy allows for covalent immobilization of the tension probes on azide-modified substrates via strain-promoted cycloaddition reaction. On the next day, the unbound DNA probes were removed with 3× PBS washes. Then, streptavidin (10 μg/ml) was added to the channels and incubated for 45 min at RT. The surfaces were cleaned with 3× PBS washes. Next, ICAM-1 ligand (5 μg/ml) was added to the surfaces, incubated for 45 min at RT, and washed thrice with 1× PBS. Surfaces were buffer-exchanged with HBSS before imaging.

Multiplex tension probes were prepared by a sequential click reaction. First, ~100 nM Atto647N tension probes were incubated with the BSA-blocked azide surface (prepared following the abovementioned protocol) overnight at RT. On the next day, the unbound probes were washed three times with 1× PBS. Then, streptavidin (10 μg/ml) was added to the channels, incubated for 45 min at RT, and washed with 3× PBS. Subsequently, N4 (5 μg/ml) was incubated with the tension probes for 45 min at RT and washed with 3× PBS. After this wash, the volume of channels was kept at ~50 μl to prevent drying, and preannealed Cy3B tension probes were added to the channel and incubated overnight at RT. On the next day, the surfaces were washed with 3× PBS. The channels were washed with 3× PBS. Then, streptavidin (10 μg/ml) was added to the channels and incubated for 45 min at RT. The surfaces were cleaned with 1× PBS washes. Next, ICAM-1 ligand (5 μg/ml) was added to the surfaces, incubated for 45 min at RT, and washed thrice with 1× PBS. Surfaces were buffer-exchanged with HBSS before imaging.

### Preparation of conventional TGT substrates

Similar to the tension probe substrate preparation, the TGT probes were assembled in 1 M NaCl by mixing the top strand (biotin-labeled strand, 200 nM) and bottom strand (dibenzocyclooctyne (DBCO)-labeled strand, 200 nM) in a 1:1 ratio. The mixture was heat-annealed at 95°C for 5 min and then cooled to 25°C over a 30-min duration. The assembled TGT (~50 μl) was added to the channels and incubated overnight at RT. Biotinylated ICAM-1 or biotinylated OVA-N4 (5 μg/ml) was anchored on the TGT surfaces in the same way as mentioned in the previous section.

### Preparation of substrates copresenting ICAM-1 TGT and surface-immobilized antigen

No. 1.5H glass coverslips (ibidi) were sequentially sonicated in Milli-Q water (18.2 megohms cm^−1^) and 200 proof ethanol, 10 min each. The glasses were rinsed copiously with Milli-Q water and immersed in freshly prepared piranha solution (3:1 sulfuric acid:H_2_O_2_) for 30 min to remove organic residues from and activate hydroxyl groups on glasses (CAUTION: Piranha is highly reactive and explosive on contact with organics!). The cleaned substrates were rinsed with Milli-Q water in a 200-ml beaker at least six times and further washed with ethanol thrice. Slides were then transferred to a 200-ml beaker containing 3% APTES in ethanol for 1 hour, washed with ethanol thrice, and thermally cured in an oven (~110°C) for 15 min. The slides were then mounted to six-channel microfluidic cells (Sticky-Slide VI 0.4, ibidi). To each channel, a solution of ~50 μl of NHS-PEG_4_-azide (10 mg/ml) and varying amount (0.001 to 1 mg/ml) of NHS-PEG_4_-biotin (ratio of azide:biotin = 10:1 to 10,000:1) in 0.1 M NaHCO_3_ (pH 9) was added and incubated for 1 hour.

The channels were washed with 1 ml of Milli-Q water thrice, and the remaining water in the channels was removed by pipetting. The surfaces were further blocked with 0.1% BSA in 1× PBS for 30 min and washed thrice with 1× PBS, and ~50 μl of solution was kept inside the channel to prevent drying. Subsequently, streptavidin (10 μg/ml) was added to the channels and incubated for 45 min at RT. The surfaces were cleaned with 3× PBS washes. Next, OVA-N4 ligand (5 μg/ml) or OVA-APL (e.g., Q4, T4, or V4) was added to the surfaces, incubated for 45 min at RT, and washed thrice with 1× PBS. Preassembled 100 nM TGT probe (12 or 56 pN) was then added to the channels and incubated overnight at RT. On the next day, unbound TGT probe was washed away with 3× PBS. Then, streptavidin (10 μg/ml) was added to the channels and incubated for 45 min at RT. The surfaces were cleaned with 3× PBS washes. Next, ICAM-1 (5 μg/ml) was added to the surfaces, incubated for 45 min at RT, and washed thrice with 1× PBS. Surfaces were buffer-exchanged with HBSS before imaging.

### Preparation of turn-on TGT substrates

The turn-on TGT substrates were prepared using identical protocol listed in the “Preparation of DNA hairpin-based molecular tension probes on glass surfaces” section until the APTES functionalization step. After functionalization of APTES, the slides were incubated with sulfosuccinimidyl 4-(N-maleimidomethyl)cyclohexane-1-carboxylate (sulfo-SMCC, 10 mg/mL) for 1 hour, washed with ethanol thrice, and dried under nitrogen. The slides were then mounted to six-channel microfluidic cells (Sticky-Slide VI 0.4, ibidi). Subsequently, the turn-on TGT probes were assembled in 1 M NaCl by mixing the fluorophore-labeled 12- or 56-pN bottom strands (100 nM) and the quencher strand (200 nM). The mixture was heat-annealed at 95°C for 5 min and cooled down to 25°C for 30 min. One hundred microliters of the assembled probe was reacted with 10 μM Tris(2-carboxyethyl)phosphine (1 μl, stock concentration = 1 mM) for 10 min to activate the thiol group. The annealed, thiol-activated duplexes were then added to channels, incubated for 1 hour at RT, and washed thrice with 1× PBS. Then, streptavidin (10 μg/ml) was added to the substrates and incubated for 45 min at RT and washed thrice with 1× PBS. Next, biotinylated OVA-N4/ICAM-1 (5 μg/ml) was added to the substrates, incubated for 45 min at RT, and washed thrice with 1× PBS. Surfaces were buffer-exchanged with HBSS before imaging.

### Immunofluorescence staining

A total of ~1 × 10^5^ cells cultured on surfaces were fixed by 4% formaldehyde in 1× PBS for 10 min. The surfaces were gently washed thrice with 1× PBS to prevent cell detachment. Cells were then permeabilized in 0.1% Triton X-100 for 5 min and washed thrice with 1× PBS. Subsequently, 50 μl of 2% BSA was added to the surfaces and incubated overnight at 4°C (with a total volume of 100 μl inside the channel). On the next day, the surfaces were washed thrice with 1× PBS and ~50 μl of solution was kept inside the channel. Twenty microliters of the Alexa Fluor 647 Mouse Anti-ZAP70 (PY319)/Syk (PY352) was added to each channel and incubated for 1 hour at RT. Surfaces were then washed thrice with 1× PBS and buffer-exchanged with 1× HBSS before imaging.

### Image analysis

Fluorescence images were processed using the Fiji ImageJ software. Fluorescence background was subtracted. Cell spreading was measured by the total contact area of cell reflected by RICM. Fluorescence signal (e.g., tension probe signal or immunostained pY-ZAP70) was measured at the cell-substrate interface either by measuring the mean intensity (for tension probe data) or integrated intensity (for immunostaining data). Brightness and contrast of microscopy images were adjusted for presentation.

### Statistics

All experiments were conducted as at least three technical and biological replicates. All tests were performed in Prism 7 (GraphPad Software), and data were presented as means ± SEM or scatter plots showing all data points with line representing mean. Unless otherwise stated, groups were compared using Student’s *t* test.

## Supplementary Materials

### This PDF file includes:

Figs. S1 to S21 Tables S1 to S3 Notes S1 and S2

## Notes

View/request a protocol for this paper from *Bio-protocol*.
