# Supplementary material for: The magnitude of LFA-1/ICAM-1 forces fine-tune TCR-triggered T cell activation
Source: Sci Adv. 2022 Feb 25;8(8):eabg4485. doi: 10.1126/sciadv.abg4485 (PMC8880789; doi:10.1126/sciadv.abg4485)
Supplement: Supplementary file 1 — Figs. S1 to S21 Tables S1 to S3 Notes S1 and S2 [file sciadv.abg4485_sm.pdf]

Science Advances
AAAS

Supplementary Materials for

The magnitude of LFA-1/ICAM-1 forces fine-tune TCR-triggered T cell activation

Victor Pui-Yan Ma, Yuesong Hu, Anna V. Kellner, Joshua M. Brockman, Arventh Velusamy, Aaron T. Blanchard, Brian D. Evavold, Ronen Alon, Khalid Salaita*

*Corresponding author. Email: k.salaita@emory.edu

Published 25 February 2022, *Sci. Adv.* **8**, eabg4485 (2022)
DOI: 10.1126/sciadv.abg4485

The PDF file includes:

Figs. S1 to S21
Tables S1 to S3
Notes S1 and S2
Legends for movies S1 to S7

Other Supplementary Material for this manuscript includes the following:

Movies S1 to S7

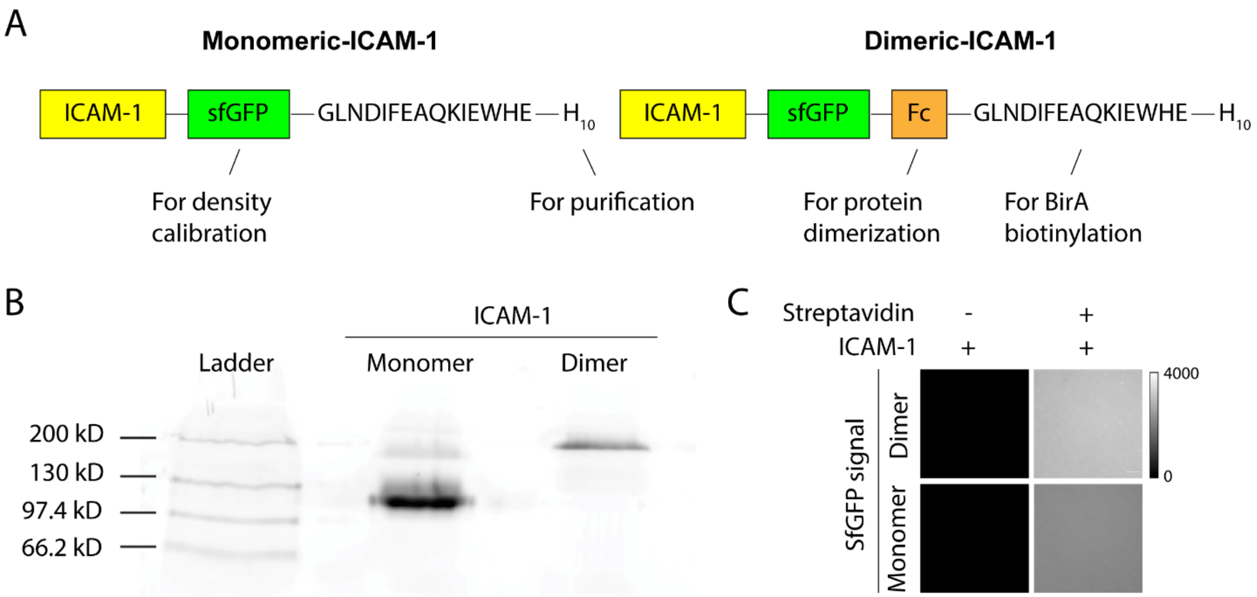

Fig. S1. Characterization of biotinylated ICAM-1 constructs. A) Functional map of the monomeric and dimeric ICAM-1s. **B)** 6% non-reducing SDS-PAGE separating gel showed estimated molecular weights of the monomeric and dimeric ICAM-1. Bands were visualized with a Tycoon imager equipped with fluorescent readout with excitation for the FITC channel. **C)** Characterizing binding of the biotinylated monomeric and dimeric ICAM-1 on SLB (99.9% DOPC/0.1% Biotin-Cap PE). As a control, streptavidin was withheld, and no non-specific binding were observed.

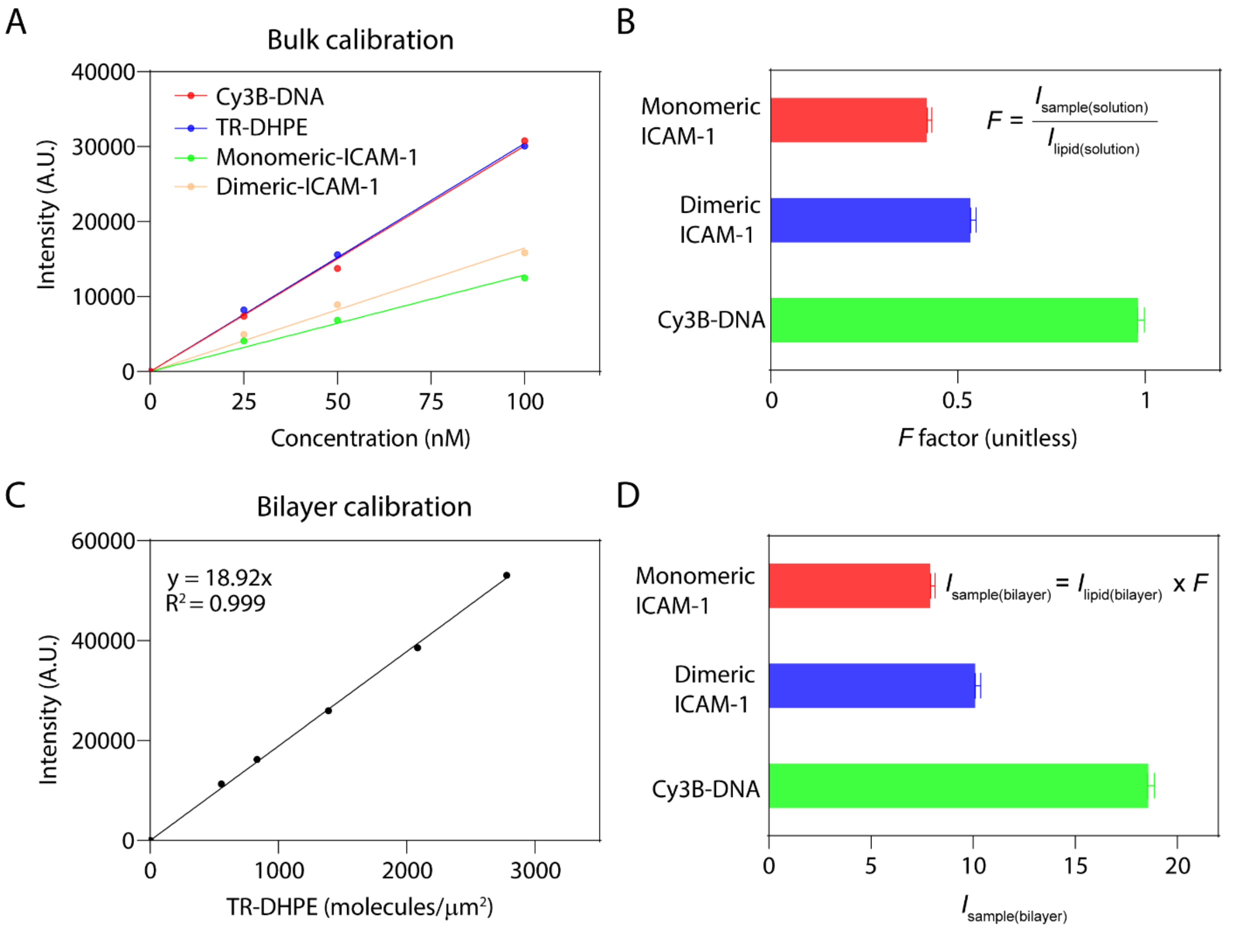

Fig. S2. Qualitative fluorescence microscopy for density calibration of tension probe/ICAM-1. A) Brightness of samples and TR-DPHE liposome were measured using fluorescence microscopy. **B)** A scaling factor *F* was generated to relate the brightness of sample to the TR-DPHE by from the ratio of slopes from solution calibrations. **C)** A TR-DHPE bilayer calibration curve with known molecular densities. **D)** *F* factor was used to scale the slope of the TR bilayer calibration to estimate the relation between the sample intensity on a surface and its surface density. Density of ICAM-1 and Cy3B-tension probe used in respective experiments are summarized in **Table S3**.

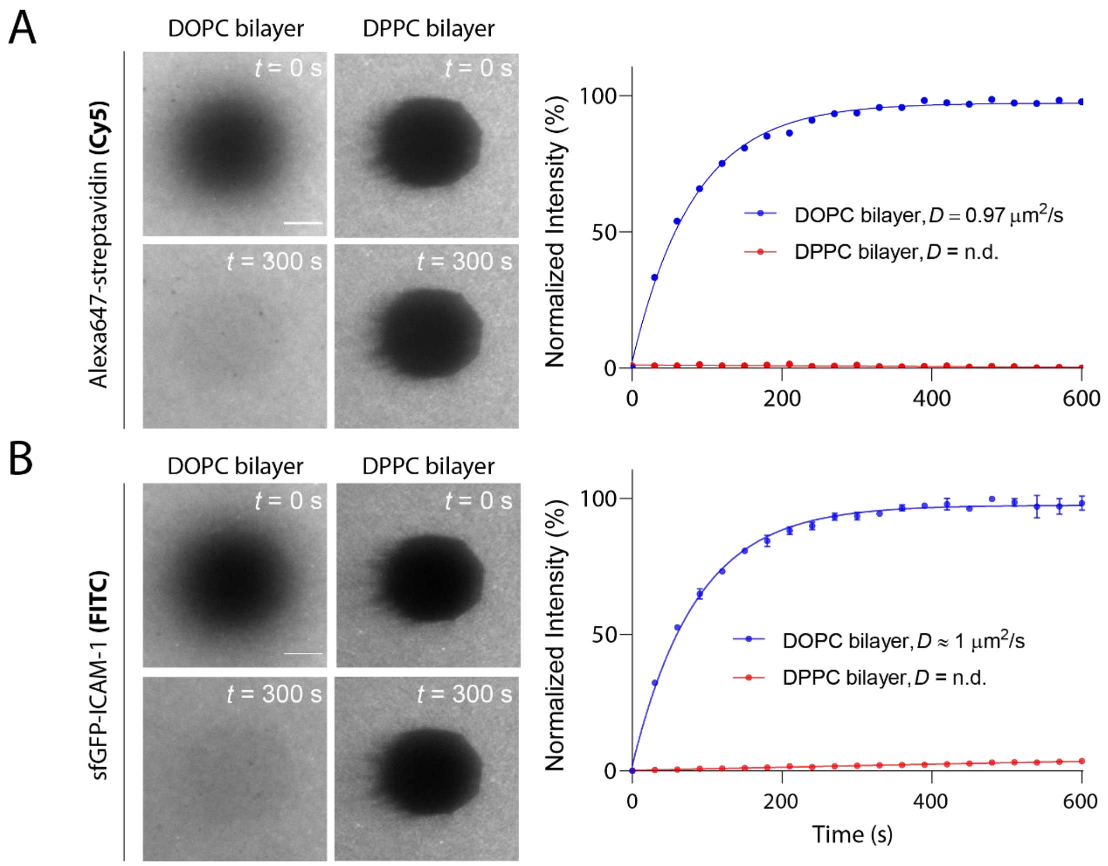

Fig. S3. SLB characterization. Fluorescence Recovery After Photobleaching (FRAP) experiment showing the lateral mobility of **A**) Alexa647 tagged streptavidin and **B**) dimeric ICAM-1 can be manipulated using bilayers with different fluid-to-gel transition temperatures (99 mol% DOPC or DPPC + 0.1mol% biotinyl-Cap PE). Right are FRAP plots showing recovery of GFP or Alexa647 fluorescence over the course of 600 s. The Lateral diffusion coefficients (D) were calculated by: $D = w^2/4t_{1/2}$, where w is the radius of the Gaussian bleaching area and $t_{1/2}$ is the time for 50% recovery obtained from the fit. Experiments were performed at 25 °C. Scale bar = 10 μm.

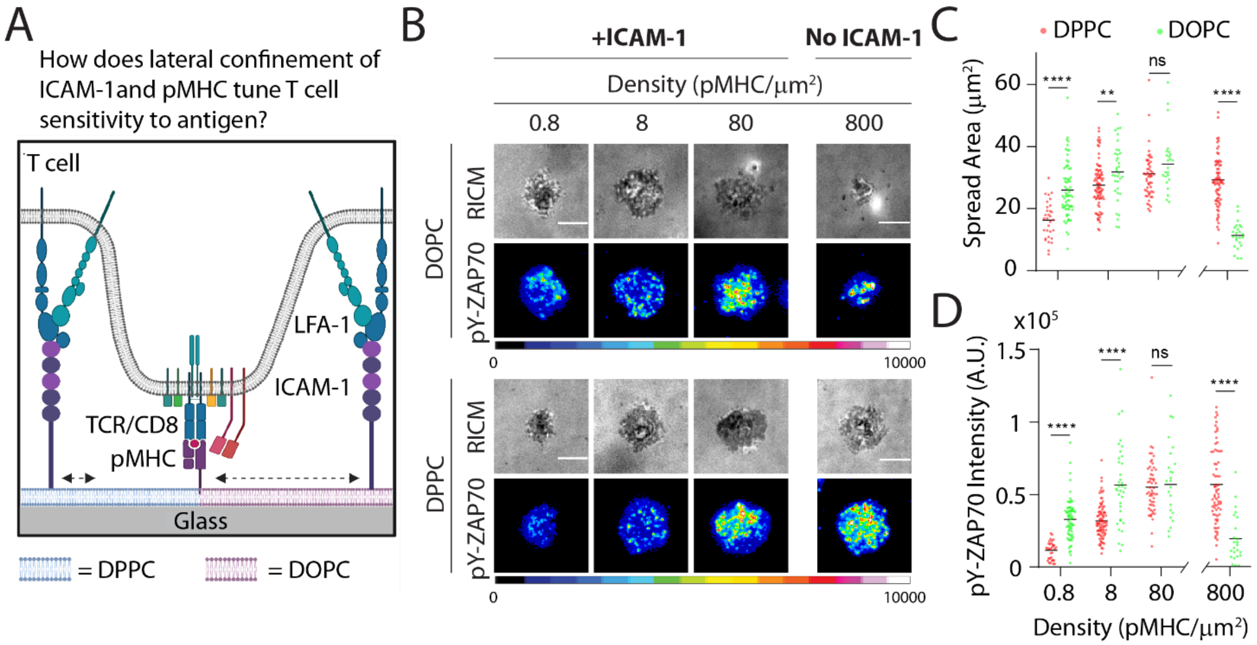

Fig. S4. Both DOPC and DPPC stimulatory bilayers trigger T cell spreading and activation. **A)** *In vitro* reconstitution of TCR/pMHC and LFA-1/ICAM-1 interactions in lipid bilayers with either low (DPPC) or high (DOPC) lateral mobility. **B)** RICM and immunofluorescence images of naïve OT-1 cells interacting with DOPC- or DPPC-SLBs co-presenting the ICAM-1 and OVA-N4 after ~30 min of plating. Cells were stained with Alexa 647-pY-ZAP70 antibody. **C)** Plot quantifying the spread area of cells interacting with stimulatory bilayers with indicated pMHC density. **D)** Plot showing pY-ZAP70 intensity of cells interacting with stimulatory bilayers with indicated pMHC density. Density of ICAM-1 is ~700-800 molecules/μm^2. N > 40 cells from three different experiments. Line represents mean. **P < 0.01, ****P < 0.0001. Scale bar = 5 μm.

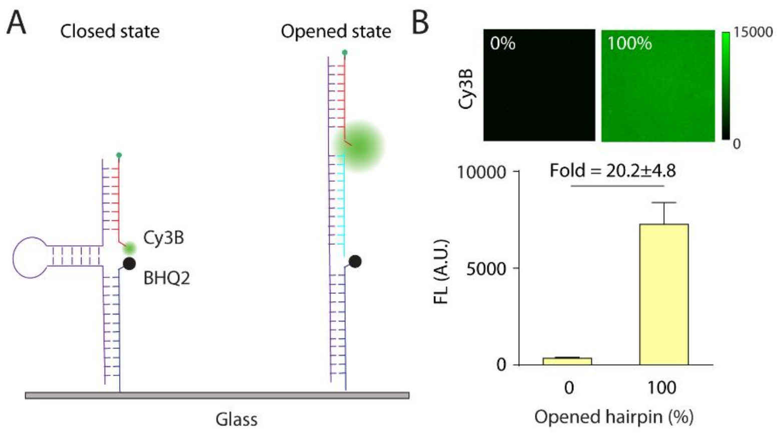

Fig. S5. Quantifying probe quenching efficiency. A) Artificial opening of DNA force probes using a full complement (teal) that binds to the hairpin stem-loop, and this hybridization subsequently leads to de-quenching of the dye. **B)** Quantification of the fluorescence intensity of the "closed" and "opened" DNA probes immobilized on the surface via copper-free click reaction.

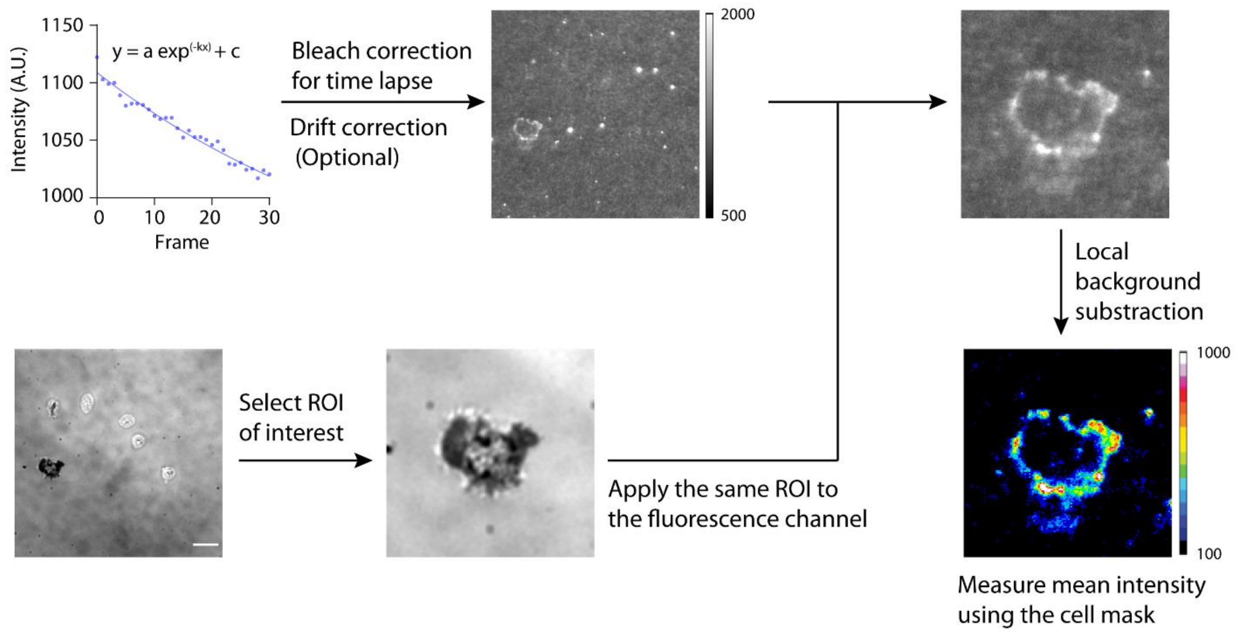

Fig. S6. Image analysis pipeline to process the force signal. Briefly, time-lapse tension videos were subjected to photobleaching correction and drift correction. Then, a region of ROI containing at least a cell was isolated in both the tension and RICM images. From the cropped ROI, a manual local background subtraction was performed to remove signals contributed from DNA probes that were not subjected to cell pulling. The corrected image displaying tension signals were isolated using cell masks.

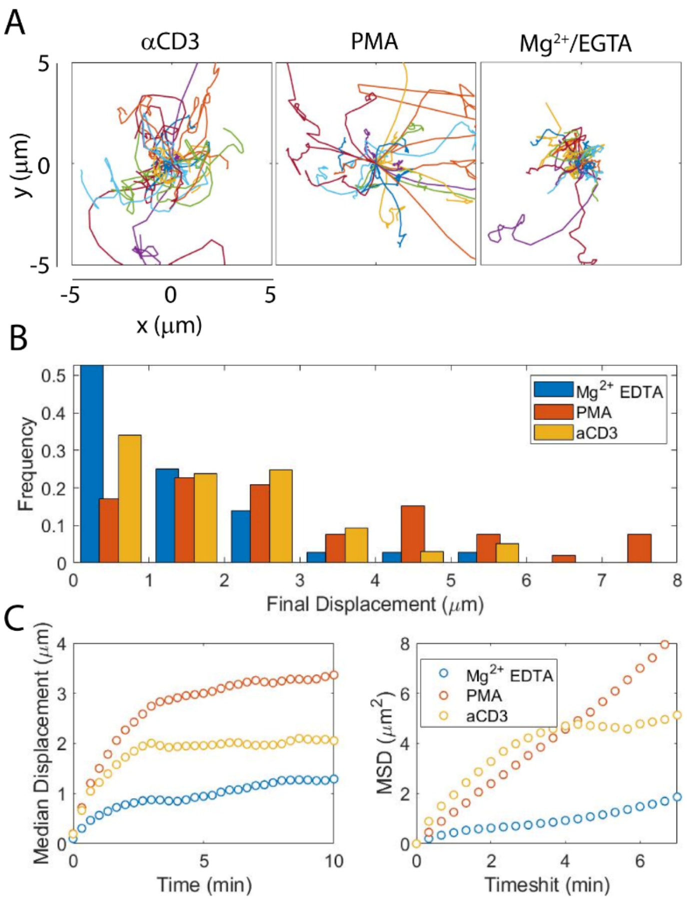

Fig. S7. Migration analysis on *in situ* stimulated cells seeded on the dimeric ICAM-1 tension probes. A) 2D displacement plot showing ~30 tracks of cell movement in 10 min. Starting positions are normalized to the origin. **B)** Plots showing final displacements of cells from the original position. **C)** Median displacement and MSD plots showing the migration behaviour of stimulated cells. N > 50 cells from three independent experiments.

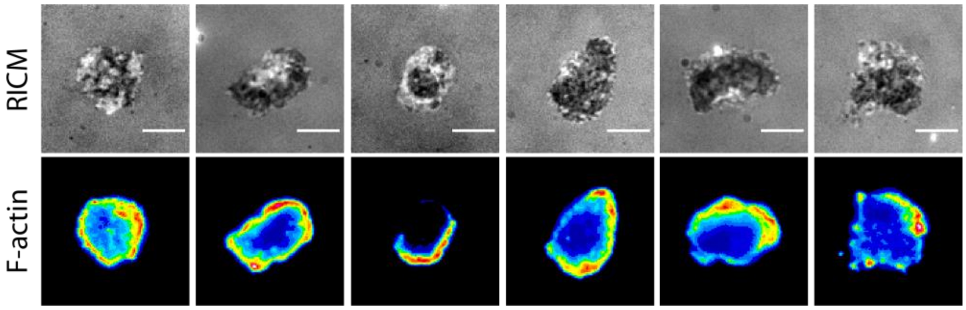

Fig. S8. *F*-actin staining. aCD3 primed OT-1 cells cultured on 4.7 pN ICAM-1 tension probe for 10 min and immunostained for *F*-actin. This result showed *F*-actin was mainly distributed at the cell periphery where LFA-1 generated significant tension.

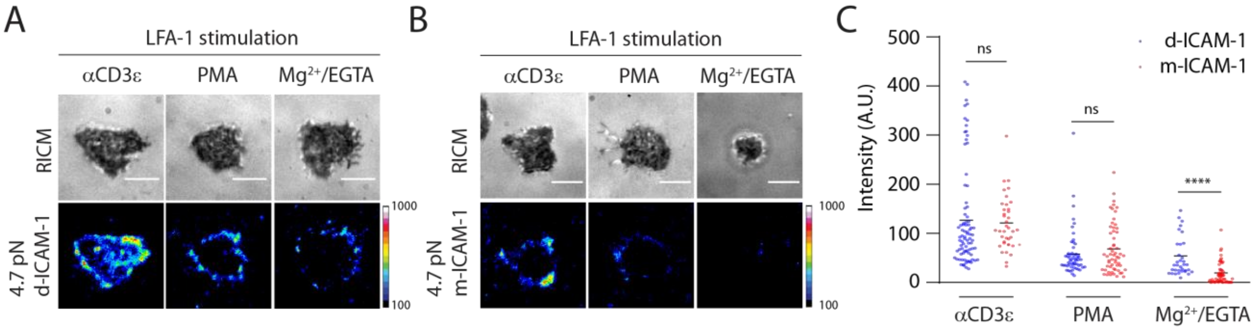

Fig. S9. Force transmitted across LFA-1/ICAM-1 complex when naïve OT-1 cells were activated with different mitogetic stimuli. A) RICM and tension images showing *in situ* primed cells with different agents on dimeric ICAM-1 tension probe surfaces after ~15-20 min. **B)** RICM and tension images showing *in situ* primed cells with different agents on monomeric ICAM-1 tension probe surfaces after ~15-20 min**. C)** Plot qualifying the mean tension intensity underneath the cell-substrate contact. N > 40 cells from three independent experiments. ****P < 0.001. Scale bars = 5 µm.

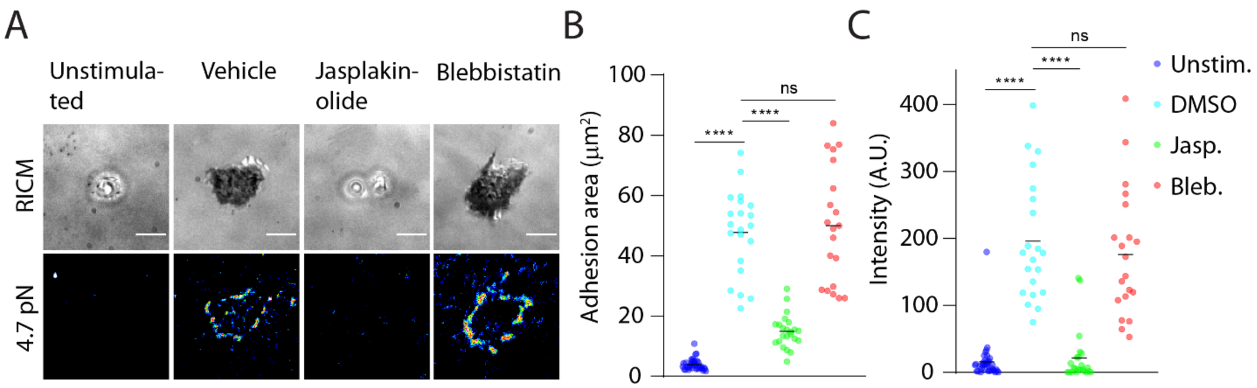

Fig. S10. Impact of cytoskeletal drugs on LFA-1 mediated adhesion, spreading and force generation on dimeric ICAM-1 tension probe substrates. Naïve OT-1 cells were pre-treated with vehicle control (0.1% DMSO, 30 min), 0.5 µM Jasplakinolide (1 h), or 50 µM blebbistatin (30 min). Prior to cell seeding, 10 µg/mL aCD3 was infused with drug treated cells. **A)** Representative RICM and tension images of cells seeded on the ICAM-1 tension probe substrates for ~20 min. **B)** Plot showing adhesion area for control cells and inhibitor treated cells. **C)** Plot showing mean tension intensity for control cells and inhibitor treated cells. ****P <0.0001.

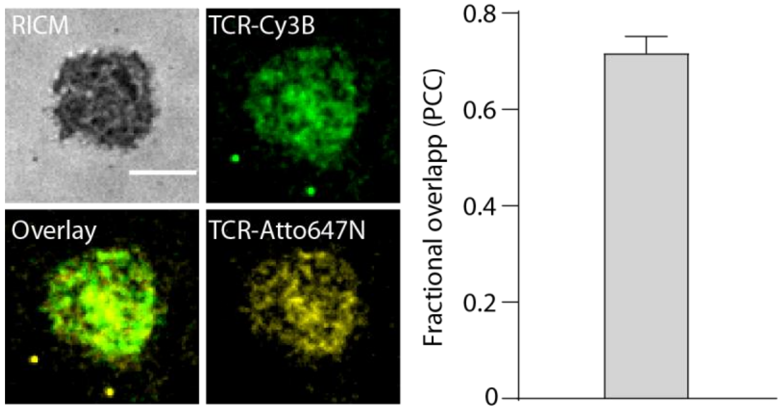

Fig. S11. Positive control for multiplexed tension colocalization. RICM and tension images of naïve CD8+ OT-1 cells seeded on the multiplexed tension probe substrates where both the Cy3B and Atto647N probes presented OVA-N4 ligand. The force patterns in both channels were highly colocalized, with an average PCC = 0.72±0.03, indicating moderate to high overlapping. Scale bar = 5 µm. N = 10 cells.

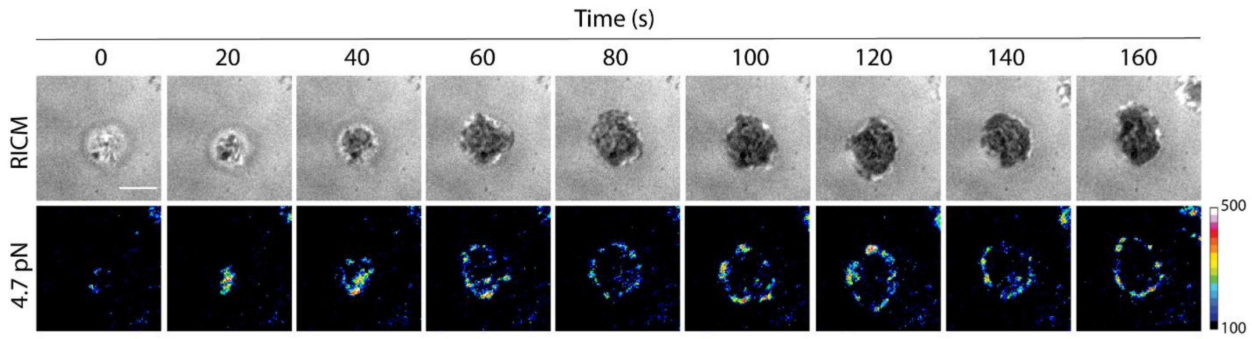

Fig. S12. **Time lapse images showing early T cell engagement and spreading on ICAM-1 tension sensor surface co-presenting OVA N4-antigen.** LFA-1 transmitted forces on tension probe substrate ($F_{1/2}$ = 4.7 pN) was imaged in the Cy3B channel and adhesion was measured using RICM. Scale bar = 5 µm.

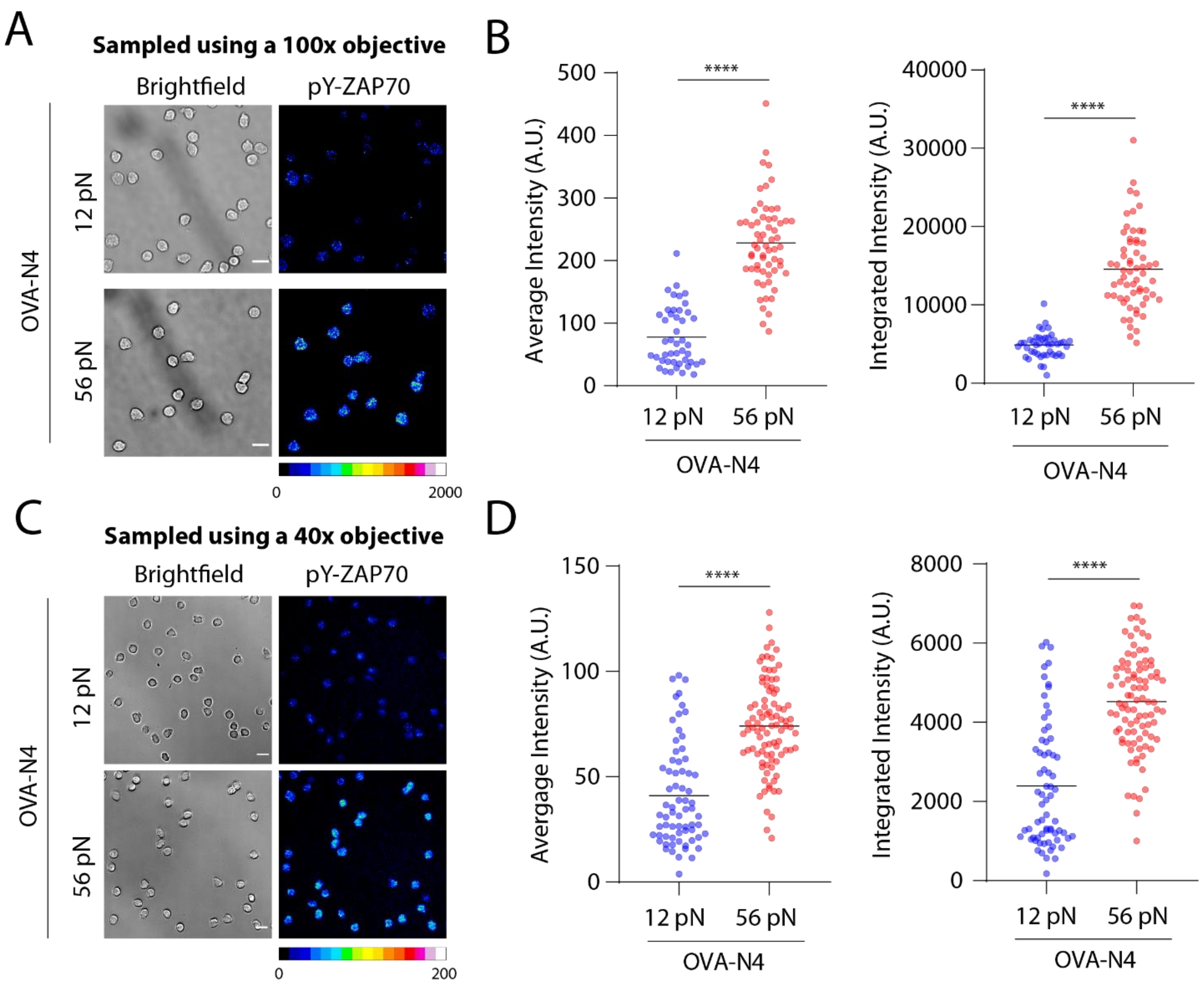

Fig. S13. TCR-pMHC tension magnitude enhances TCR activation. A) Representative RICM and pY-ZAP70 immunofluorescence image (using a 100x oil objective) of naïve OT-1 cells seeded on 12 pN TGT or 56 pN TGT substrates for 1h. The surfaces presented OVA-N4 pMHC. **B)** Quantification both average and integrated pY-ZAP70 intensity of cells seeded on 12 pN or 56 pN TGT substrates using cell masks with same region of interest. N > 30 cells from three independent experiments. ****P < 0.0001. **C)** Representative brightfield and immunofluorescence images (imaged using a 40x objective) of naïve OT-1 cells seeded on 12 pN TGT or 56 pN TGT substrates for 1h. **D)** Quantification both average and integrated pY-ZAP70 intensity of cells seeded on 12 pN or 56 pN TGT substrates using cell masks with same region of interest. N > 50 cells from three independent experiments. **** P < 0.0001. Scale bars = 10 µm. For all experiments, Alexa-647 phospho-ZAP70 antibody was used to stain the cells and BF-defined ROI to compare the average intensity versus integrated intensity of pY-ZAP70 of cells seeded on 12 pN or 56 pN N4 TGT surfaces. This confirms integrated intensity of pY-ZAP70 is a valid readout for the degree of early T cell activation.

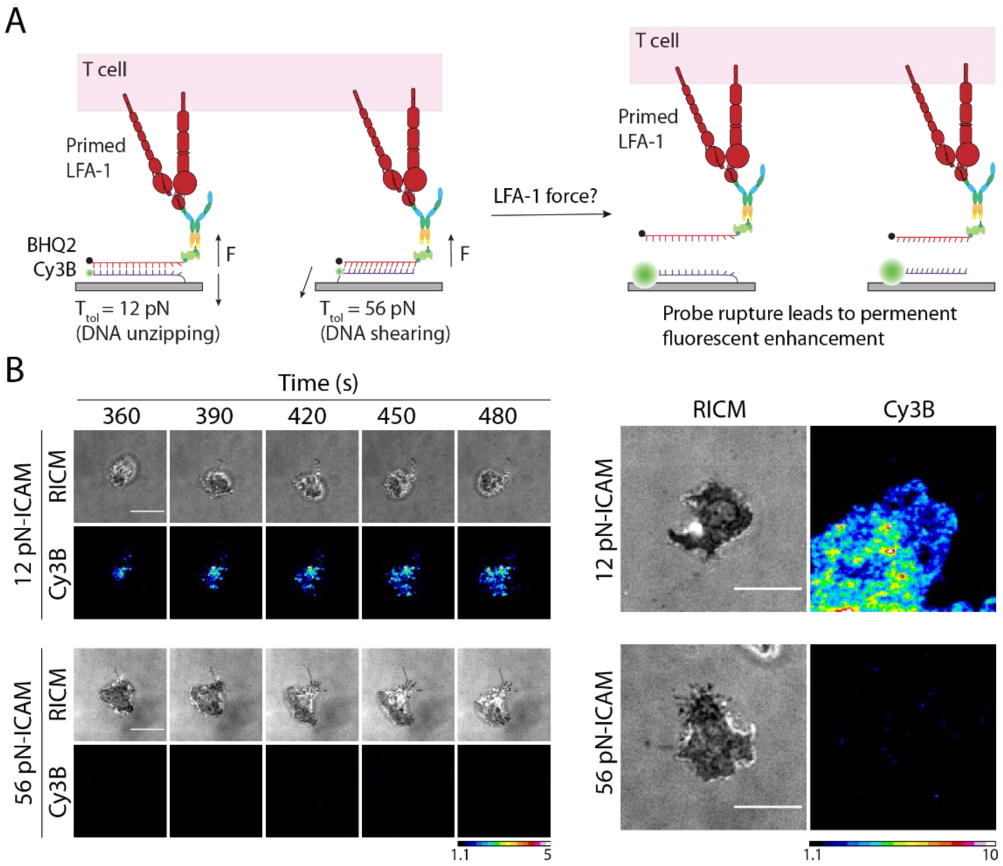

Fig. S14. Mapping LFA-1/ICAM-1 peak tension using turn-on TGT probes. A) Schematic showing the design of turn-on TGT. **B)** Representative RICM time-lapse images and the corresponding turn-on TGT signal of αCD3 primed OT-1 cells. These cells were seeded on 12 pN ICAM-1-TGT or 56 pN ICAM-1 TGT substrates, and we started recording the time-lapse movies at ~5 min. **C**) αCD3 primed cells on ICAM-1 TGTs at t = ~30 min after seeding. Tension Images were normalized to the probe background for better visualization. Calibration bar represents fold change in fluorescence over background. Scale bar = 5 µm.

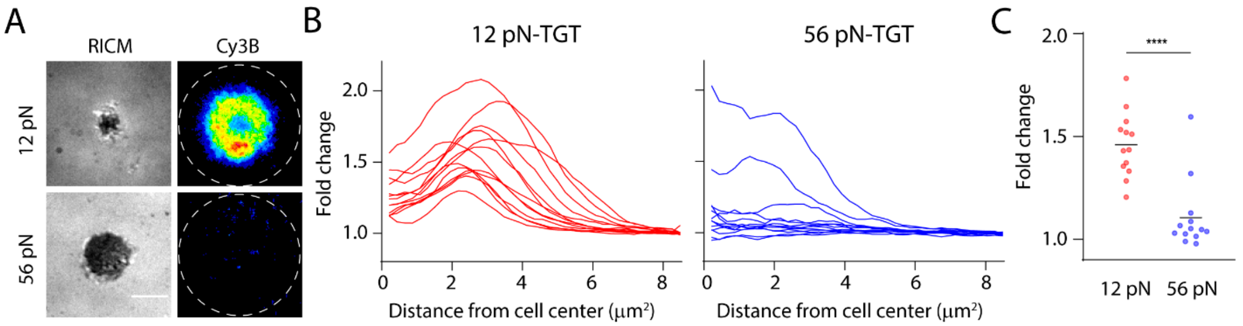

Fig. S15. Turn-on TGT signal is specific to receptor-ligand interaction illustrated using a cognate antigen control. A) Representative RICM and turn-on TGT signal of cells seeded on 12 pN N4-TGT or 56 pN N4-TGT substrates for ~30 min. Tension images were normalized to background fluorescence. **B)** Single cell radial profiles of the force application history of cells seeded on 12 pN or 56 pN TGT substrates. **C)** Quantification of the fold enhancement of the fluorescence underneath the cells. N = ~15-20 cells. **** P < 0.0001.

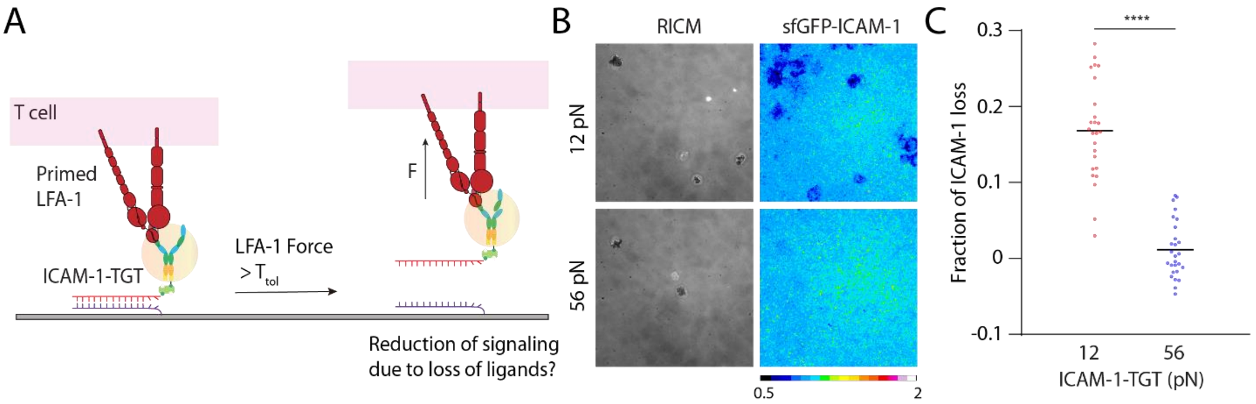

Fig. S16. Quantifying ICAM-1 mechanical dissociation as function of TGT tolerance. A) Schematic showing T cell-mediated probe rupture leads to loss of sfGFP-ICAM-1 fluorescence. **B)** Representative whole field RICM fluorescence images (512 x 512 pixels) of αCD3ε primed OT-1 cells on 12 or 56 pN ICAM-1 TGT surfaces for ~30 min. **C)** Quantification of the fraction of ICAM-1 loss of darkened area nearby or underneath the cells. N = ~15-20 cells. **** P < 0.0001.

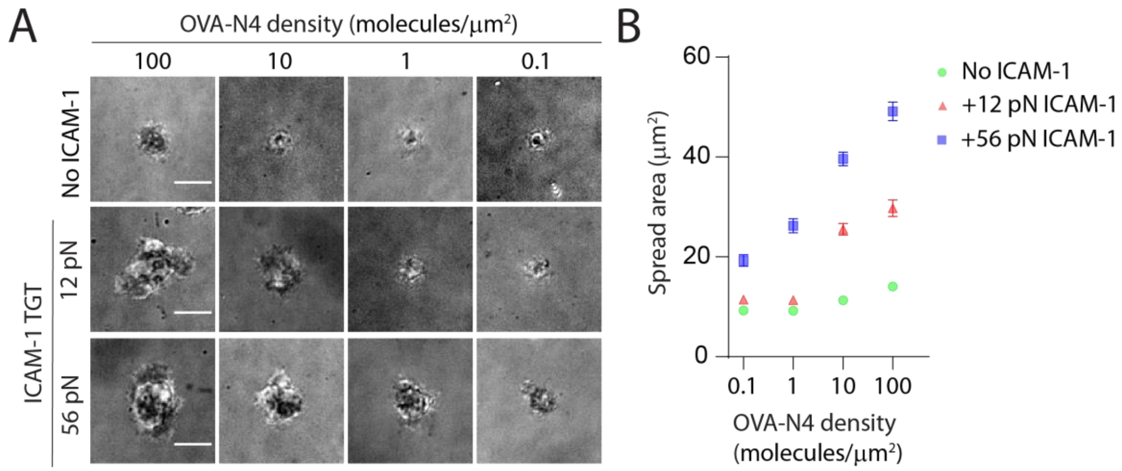

Fig. S17. **A)** Representative RICM images of cells seeded on surfaces coated with immobilized OVA-N4, immobilized OVA-N4 and 12 pN ICAM1-TGT, or immobilized OVA-N4 and 56 pN ICAM-1-TGT. Antigen densities were varied from ~0.1 to ~100 molecules/μm^2. Scale bar = 5 μm. **B)** Quantification of spread area of cells seeded on OVA-N4 only, OVA-N4 and 12 pN ICAM-1 TGT or OVA-N4 and 56 pN ICAM-1 TGT substrates.

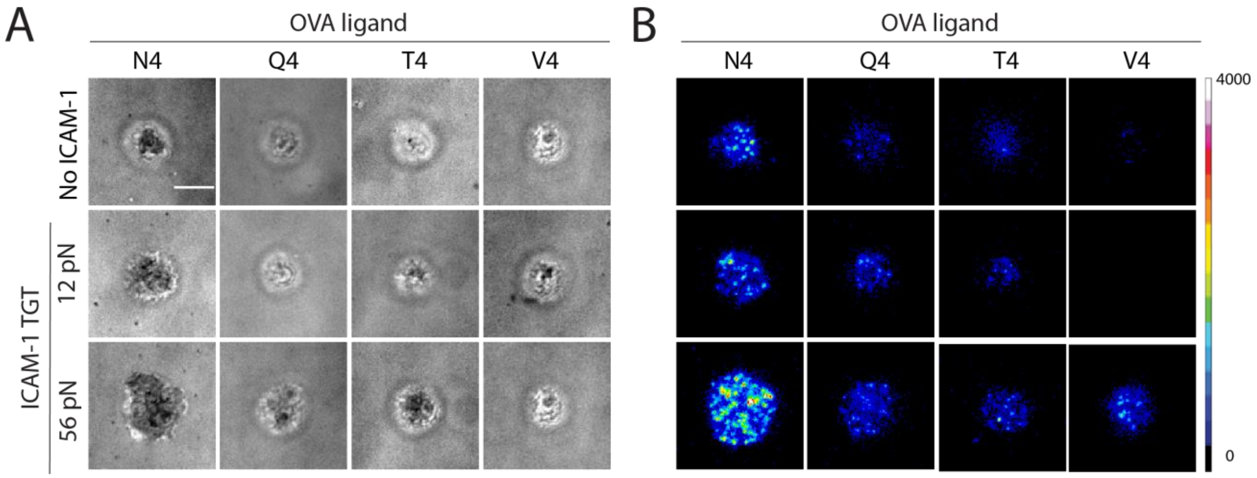

Fig. S18. A) Representative RICM and **B)** Immunostained pY-ZAP70 images of OT-1 cells seeded on surfaces coated with immobilized antigen, immobilized antigen and 12 pN ICAM1-TGT, or immobilized antigen and 56 pN ICAM-1-TGT. Antigen densities was fixed at ~1 molecule/μm^2. Scale bar = 5 μm**. B)** Quantification of spread area of cells seeded on antigen only, antigen and 12 pN ICAM-1 TGT or antigen and 56 pN ICAM-1 TGT substrates. A panel of antigens was tested including the parental N4 (SIIQFEKL), and the altered peptide ligands: Q4 (SIIQFEKL); T4 (SIITFEKL); and V4 (SIIVFEKL).

Table S1. Annotated amino acid sequences of the soluble ICAM-1 constructs.

Monomeric ICAM-1	M A S T R A K P T L P L L L A L V T V V I P G P G D A Q V S I H<br>P R E A F L P Q G G S V Q V N C S S S C K E D L S L G L E T Q W<br>L K D E L E S G P N W K L F E L S E I G E D S S P L C F E N C G T<br>V Q S S A S A T I T V Y S F P E S V E L R P L P A W Q Q V G K D<br>L T L R C H V D G G A P R T Q L S A V L L R G E E I L S R Q P V<br>G G H P K D P K E I T F T V L A S R G D H G A N F S C R T E L D<br>L R P Q G L A L F S N V S E A R S L R T F D L P A T I P K L D T P<br>D L L E V G T Q Q K L F C S L E G L F P A S E A R I Y L E L G G<br>Q M P T Q E S T N S S D S V S A T A L V E V T E E F D R T L P L<br>R C V L E L A D Q I L E T Q R T L T V Y N F S A P V L T L S Q L<br>E V S E G S Q V T V K C E A H S G S K V V L L S G V E P R P P T<br>P Q V Q F T L N A S S E D H K R S F F C S A A L E V A G K F L F<br>K N Q T L E L H V L Y G P R L D E T D C L G N W T W Q E G S Q<br>Q T L K C Q A W G N P S P K M T C R R K A D G A L L P I G V V<br>K S V K Q E M N G T Y V C H A F S S H G N V T R N V Y L T V L<br>Y H S Q N N S K G E E L F T G V V P I L V E L D G D V N G H K F<br>S V R G E G E G D A T N G K L T L K F I C T T G K L P V P W P T<br>L V T T L T Y G V Q C F S R Y P D H M K R H D F F K S A M P E<br>G Y V Q E R T I S F K D D G T Y K T R A E V K F E G D T L V N R<br>I E L K G I D F K E D G N I L G H K L E Y N F N S H N V Y I T A D<br>K Q K N G I K A N F K I R H N V E D G S V Q L A D H Y Q Q N T<br>P I G D G P V L L P D N H Y L S T Q S V L S K D P N E K R D H M<br>V L L E F V T A A G I T H G M D E L Y K G L N D I F E A Q K I E<br>W H E G G G G S H H H H H H H H H H
Dimeric ICAM-1	M A S T R A K P T L P L L L A L V T V V I P G P G D A Q V S I H<br>P R E A F L P Q G G S V Q V N C S S S C K E D L S L G L E T Q W<br>L K D E L E S G P N W K L F E L S E I G E D S S P L C F E N C G T<br>V Q S S A S A T I T V Y S F P E S V E L R P L P A W Q Q V G K D<br>L T L R C H V D G G A P R T Q L S A V L L R G E E I L S R Q P V<br>G G H P K D P K E I T F T V L A S R G D H G A N F S C R T E L D<br>L R P Q G L A L F S N V S E A R S L R T F D L P A T I P K L D T P<br>D L L E V G T Q Q K L F C S L E G L F P A S E A R I Y L E L G G<br>Q M P T Q E S T N S S D S V S A T A L V E V T E E F D R T L P L<br>R C V L E L A D Q I L E T Q R T L T V Y N F S A P V L T L S Q L<br>E V S E G S Q V T V K C E A H S G S K V V L L S G V E P R P P T<br>P Q V Q F T L N A S S E D H K R S F F C S A A L E V A G K F L F<br>K N Q T L E L H V L Y G P R L D E T D C L G N W T W Q E G S Q<br>Q T L K C Q A W G N P S P K M T C R R K A D G A L L P I G V V<br>K S V K Q E M N G T Y V C H A F S S H G N V T R N V Y L T V L<br>Y H S Q N N S K G E E L F T G V V P I L V E L D G D V N G H K F<br>S V R G E G E G D A T N G K L T L K F I C T T G K L P V P W P T<br>L V T T L T Y G V Q C F S R Y P D H M K R H D F F K S A M P E<br>G Y V Q E R T I S F K D D G T Y K T R A E V K F E G D T L V N R<br>I E L K G I D F K E D G N I L G H K L E Y N F N S H N V Y I T A D<br>K Q K N G I K A N F K I R H N V E D G S V Q L A D H Y Q Q N T

	P I G D G P V L L P D N H Y L S T Q S V L S K D P N E K R D H M V L L E F V T A A G I T H G M D E L Y K P K S C D K T H T C P P C P A P E L L G G P S V F L F P P K P K D T L M I S R T P E V T C V V V D V S H E D P E V K F N W Y V D G V E V H N A K T K P R E E Q Y N S T Y R V V S V L T V L H Q D W L N G K E Y K C K V S N K A L P A P I E K T I S K A K G Q P R E P Q V Y T L P P S R D E L T K N Q V S L T C L V K G F Y P S D I A V E W E S N G Q P E N N Y K T T P P V L D S D G S F F L Y S K L T V D K S R W Q Q G N V F S C S V M H E A L H N H Y T Q K S L S L S P G K G L N D I F E A Q K I E W H E G G G G S H H H H H H H H H H

Orange: mouse ICAM-1 (Ig superfamily domains 1-5)
Green: superfolder GFP
Grey: Fc IgG1 heavy chain constant region
Teal: Avitag sequence for BirA biotinylation
Purple: His_{10} tag for Ni-NTA column purification

Table S2. Density of dimeric ICAM-1/DNA tension probe on different substrates

**Name**	**Density (molecules/μm^2)**[a]
Dimeric ICAM-1 on DOPC bilayer	~780
Dimeric ICAM-1 on DPPC bilayer	~770
Cy3B-hairpin probe (using 100% unquenched probes for estimation)	~1000
Dimeric ICAM-1 on 12 pN TGT	~400
Dimeric ICAM-1 on 56 pN TGT	~500

[a] Density of the dimeric-ICAM-1 or DNA on a substrate is estimated using the factor [$I_{\text{sample(bilayer)}}$] calculated in **Figure S3D**. Since these molecules attached to only the upper leaflet of bilayer or the DNA probe is attached on the glass coverslip, unlike the Texas Red lipid probe which is presented on both bilayer leaflets. A factor of 2 is used to "correct" the protein density.

$$Density = \frac{2 \times\ Intensity}{I_{\text{sample(bilayer)}}}$$

where intensity is the surface fluorescent signal obtained with the same CCD camera setting as the lipid calibration experiment, and $I_{\text{sample(bilayer)}}$ is defined in **Fig. S2D**.

Table S3. Oligonucleotide sequences used in this study

**Name**	**Sequence (5' to 3')**
A21B	/5AmMC6/ - CGC ATC TGT GCG GTA TTT CAC TTT - /3Bio/
Quencher	/5DBCON/ - TTT GCT GGG CTA CGT GGC GCT CTT - /3BHQ_2/
Hairpin ($F_{1/2}$ = 4.7 pN)[a]	GTG AAA TAC CGC ACA GAT GCG TTT GTA TAA ATG TTT TTT TCA TTT ATA CTT TAA GAG CGC CAC GTA GCC CAG C
Hairpin ($F_{1/2}$ = 19.3 pN)[a]	GTG AAA TAC CGC ACA GAT GCG TTT CGC CGC GGG CCG GCG CGC GGT TTT CCG CGC GCC GGC CCG CGG CGT TTA AGA GCG CCA CGT AGC CCA GC
4.7 pN loop complement	GTA TAA ATG AAA AAA ACA TTT ATA C
DBCO-bottom strand (TGT)	/5DBCON/GT GTC GTG CCT CCG TGC TGT G
12 pN top strand (TGT)	CAC AGC ACG GAG GCA CGA CAC /3Bio/
56 pN top strand (TGT)	/5Biosg/CA CAG CAC GGA GGC ACG ACA C
Biotin-BHQ2 (Top strand, turn-on TGT)	/5Biosg/ - CAC AGC ACG GAG GCA CGA CAC - /3BHQ2/
12 pN (Bottom strand, turn-on TGT)	/5UniAmM/ - GTG TCG TGC CTC CGT GCT GTG TTT TT - /3ThioMC3-D/
56 pN (Bottom strand, turn-on TGT)	/5ThioMC6-D/ - TTT TT/iUniAmM/ GTG TCG TGC CTC CGT GCT GTG

[a] Underline base represents the loop forming region

Supplementary Note 1: Orientation of external force does not affect the rupture force of a surface anchored DNA duplex

To test whether the rupture force of a DNA duplex is sensitive to force orientation, we used the course grain modeling software package (*75*). oxDNA2 implements a DNA model with major and minor grooves, coarse-grained at the level of single nucleotides with interactions to mimic hydrogen-bonding, stacking, chain connectivity and excluded volume. This allows the model to reproduce the thermodynamic, structural, and mechanical properties of DNA that match experimental data.

The DNA duplex can be designed with geometries that tune the rupture force: the unzipping geometry and the shearing geometry. In unzipping geometry (estimated T_{tol} = ~12 pN, **Fig. S18A**), force is applied to the nucleotide at the same end of the nucleotide anchoring to the coverslip. While in shearing geometry (estimated T_{tol} = ~56 pN, **Fig. S18B**), force is applied to the nucleotide in an orientation that is parallel to the long axis of the double helix.

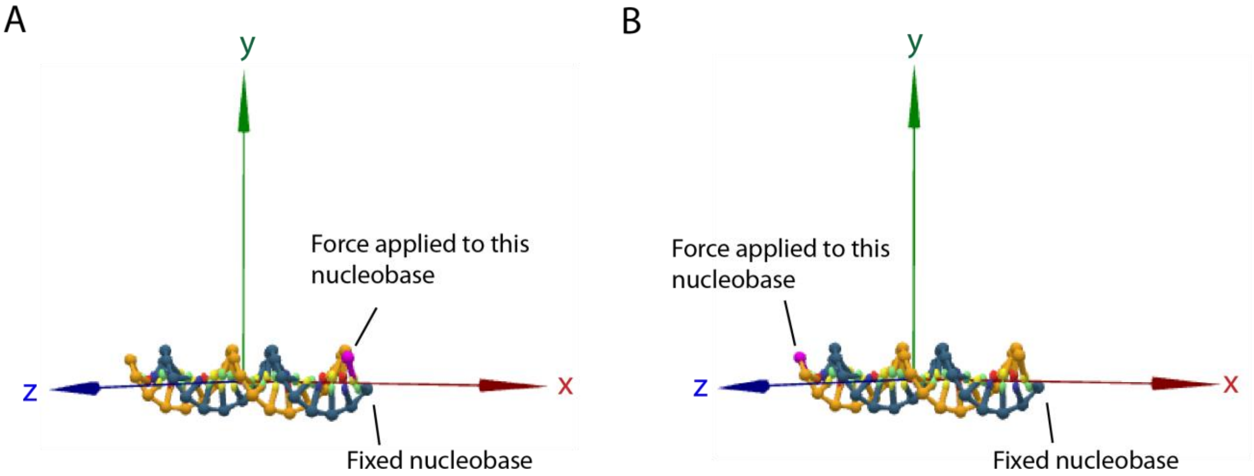

Fig. S19. Three-dimensional representations of DNA duplexes with **A)** unzipping geometry and **B)** shearing geometry**.** DNA structures are visualized using oxDNA viewer (*76*).

We ran MD simulations on oxDNA to predict the rupture force of DNA duplexes under force loading from different orientations. For each configuration, we applied force along four different orientations [(x,y,z) = (1,0,0) , (0,0,1) , (1,1,1) , (0,-1,1)] at the center of mass of the nucleotide highlighted in pink while keeping the nucleobase on the opposite strand fixed at its initial position. These 4 force orientations represent the application of pure lateral force, pure vertical force and two randomly chosen force vectors. The system is evolved as per Newtonian mechanics for a given number of steps (`newtonian_steps=103`) after which particles below a certain threshold were randomly assigned velocities and momenta from a Maxwell distribution (dictated by the value `diff_coeff=2.5`). To emulate this Brownian dynamics, `john` thermostat in oxDNA was employed. Temperature and [Na^+] were set to 37°C and 0.157M respectively to mimic *in vitro* experimental conditions. The configuration for force-extension was adopted from the protocol described by Engel and colleagues used for simulating DNA origamis under force (*77*).

For all the simulations, harmonic traps of stiffness of 11.40 pN/nm were placed at the nucleotides of interest (i.e. the pink nucleotide and the fixed nucleotide highlighted in **Fig. S19**). The effective trap stiffness can be calculated using the following equation:

$$1/k_{eff} = 1/k_1 + 1/k_2$$

An extension rate of $7.03x10^3$ nm/s was used to move the trap on the pink nucleotide along the indicated direction. A total of $2x10^9$ MD steps were run for each simulation to generate $2x10^4$ data points. To *in silico* generate a force-extension curve in each pulling direction, we extract the trap extensions from their corresponding nucleotides and then project it along the force axis. The force at a given point is caluclated by multiplying the total projected extensions with k_{eff} (5.71 pN/nm). Force is then plotted against the projected displacement of the two traps from their initial location along with a 100 point expontential moving average (EMA) of the data points using python (*78*). The rupture force was estimated by picking the peak at the point of rupture using SciPy `find_peaks` module (*79*). From the simulations, the T_{tol} of DNA duplex subjected to unzipping or shearing remains relatively constant in the four tested pulling orientations [(x,y,z) = (1,0,0) , (0,0,1) , (1,1,1) , (0,-1,1)] indicating that the direction of applied force has a minimal impact on the T_{tol} (**Fig. S20**).

The experimentally determined T_{tol} of the DNA duplex was derived from an early magnetic tweezer-based force clamp measurement by Prentiss and co-workers where the rupture force was determined by applying a constant force to shear the DNA duplex for a defined interval (e.g. 2 sec) and measuring the fraction of DNA duplex separated at that force (*80*). The constant force is incrementally increased and the T_{tol} is defined as the force that leads to 50% of duplexes dissociated within its application window, while the simulation experiments were performed by applying a defined loading velocity to the nucleotide. In a typical “force ramp” setup, the rupture force of a DNA duplex is highly dependent on the loading rate (i.e. the higher the loading rate, the higher the T_{tol}). It is also worth to note that oxDNA runs have an inherent level of stochasticity that results in small variations in force estimation between runs.

We also extracted the number of hydrogen bonds for the system throughout the course of the simulation. oxDNA considers hydrogen bonds as broken when the hydrogen bonding energy between a base pair is less than 10% of that of a fully formed hydrogen bond (**Fig. S21**).

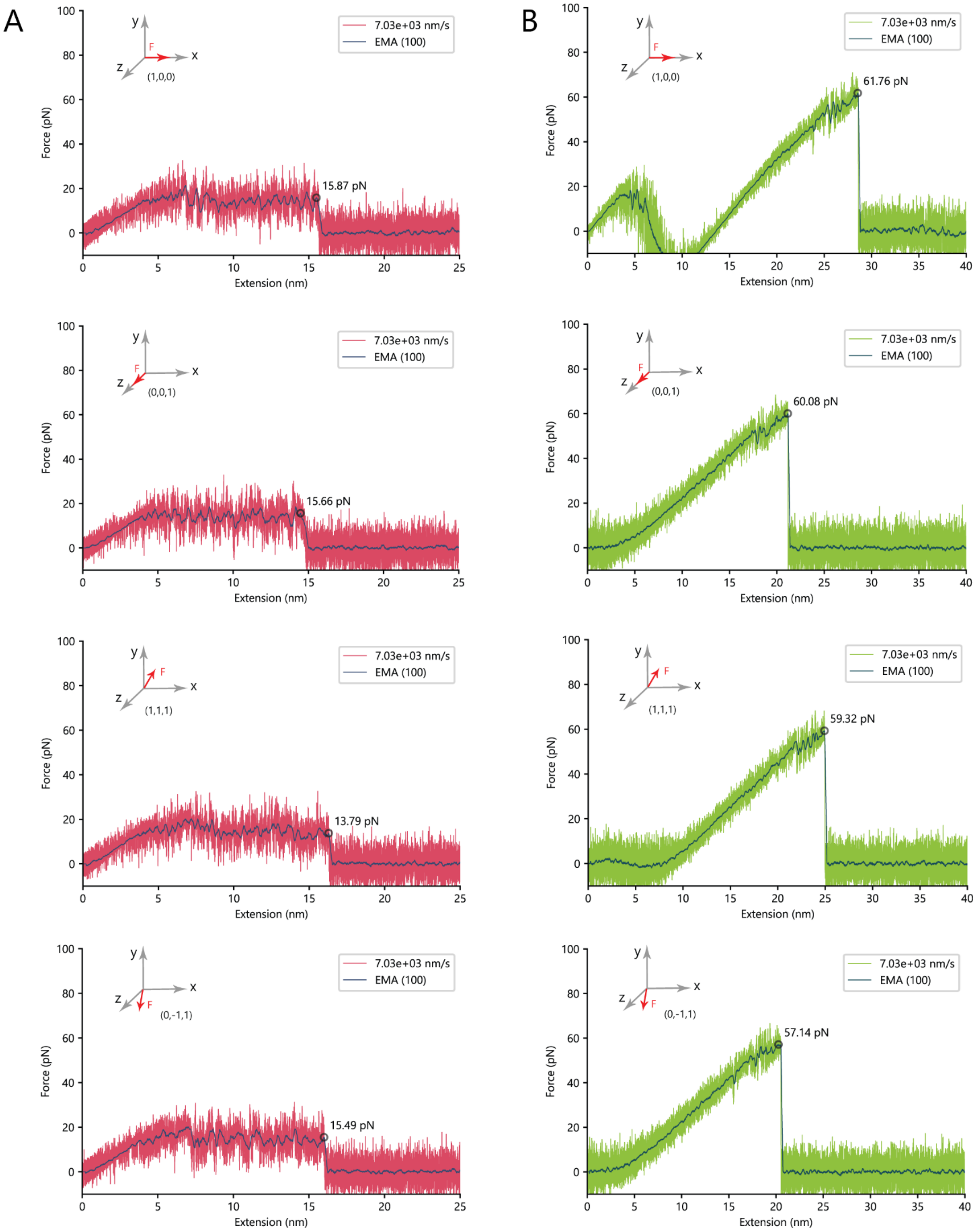

Fig. S20. OxDNA simulations of DNA rupture. Plots showing results of simulated force application along four directions (as indicated in inset) for **A)** “unzipping” TGT (red plots), and for **B)** “shearing” TGT (green plots). The dark blue curves are 100-point exponential moving average (EMA) smoothened data. Rupture force is indicated on each plot. The value of the rupture forces is independent of the pulling orientation. See **Supplementary Note 1** for detail.

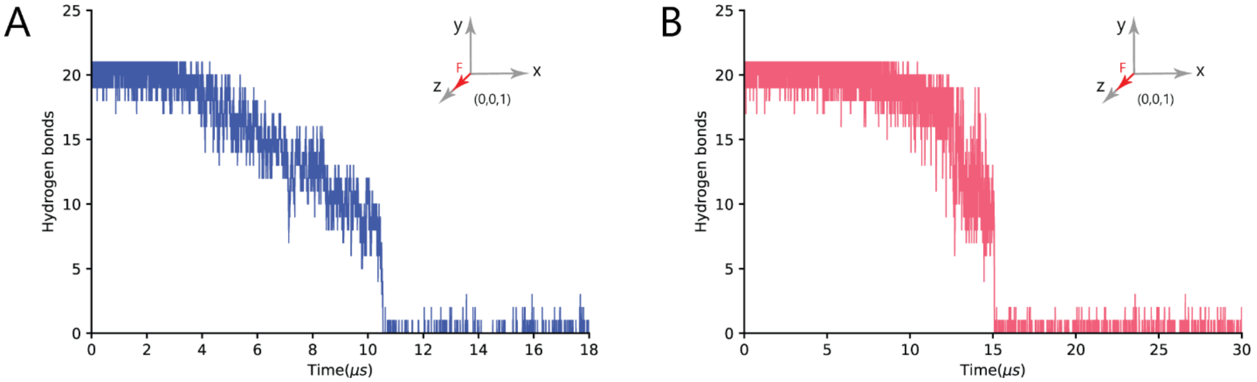

Fig. S21. **OxDNA simulations of DNA rupture.** Representative graph plotting number of DNA hydrogen bonds as a function of time for **A)** unzipping TGT and **B)** shearing TGT probes with the force application along the direction (0,0,1).

Supplementary Note 2: Cofirming pY-ZAP70 intensity is a faithful readout of early T cell activation

As a confirmatory study, we performed immunostaining experiment of pY-ZAP70 using a 40x air objective and a 100x oil objective. The axial resolution of an objective can be estimated by the following formula:

$$Resolution\ (axial) = {}^{2\lambda}/_{NA^2}$$

where λ is the wavelength of light and NA is the numerical aperture of the objective.

Considering our 100x objective has a NA of 1.49 and 40x objective has a NA of 0.65. The axial resolution for resolving a Alexa 647 molecule would be 595 nm for the 100x objective and 3,124 nm for the 40x objective. In other words, the 40x objective samples the entire thickness of the T cell and hence allows quantifying total pY-ZAP70 levels (*81*). In addition, we used the BF images to define the area occupied by the T cell. We used this BF-defined ROI to compare the average intensity versus integrated intensity of pY-ZAP70 of cells seeded on 12 pN or 56 pN N4 TGT surfaces (**Fig. S13**). We found the use of a lower magnification objective (i.e. 40x) and BF to define the cell area led to the same conclusions as those reported in the main text in **Fig. 4** (i.e. 56 pN TGT substrates support higher ZAP70 phosphorylation of T cells when compared to 12 pN substrate).

Movie captions

Movie S1. Time-lapse video showing RICM and LFA-1 tension signal of αCD3ε primed naïve OT1 cells on 4.7 pN dimeric ICAM-1 tension probe surface. Cell spreading and LFA-1 tension were imaged, after 5 min of seeding, for a duration of 10 min. Second and third channels represent unprocessed fluorescence (grey channel) and background subtracted fluorescence (colored channel) respectively. Scale bar = 10 µm.

Movie S2. Time-lapse video showing RICM and tension signals of naïve OT1 interacting with 4.7 pN dimeric ICAM-1, 4.7 pN N4 binary tension probe surface. Cell spreading, LFA-1 (green channel) and TCR (yellow channel) tension were imaged, after 5 min of seeding, for a duration of 10 min. Scale bar = 10 µm.

Movie S3. Time-lapse video showing RICM and LFA-1 tension signal of αCD3ε-primed naïve OT1 cells on 12 pN-ICAM-1 "turn-on" TGT surface. Cells were imaged, after ~5 min of seeding, for a duration of 5 min. Scale bar = 10 µm.

Movie S4. Time-lapse video showing RICM and LFA-1 tension signal of αCD3ε-primed naïve OT1 cells on 56 pN-ICAM-1 "turn-on" TGT surface. Cells were imaged, after ~5 min of seeding, for a duration of 5 min. Scale bar = 10 µm.

Movie S5. Time-lapse video showing RICM and TCR tension signal of naïve OT1 cells on 12 pN-N4 "turn-on" TGT surface. Cells were imaged, after ~5 min of seeding, for a duration of 5 min. Scale bar = 10 µm.

Movie S6. Time-lapse video showing RICM and TCR tension signal of naïve OT1 cells on 56 pN-N4 "turn-on" TGT surface. Cells were imaged, after ~5 min of seeding, for a duration of 5 min. Scale bar = 10 µm.

Movie S7. Representative time-lapse movies showing the OxDNA modeling of DNA mechanical rupture.
